# Effects of Antihypertensive Drugs Use on Risk and Prognosis of Colorectal Cancer: A Meta-Analysis of 37 Observational Studies

**DOI:** 10.3389/fphar.2021.670657

**Published:** 2022-01-11

**Authors:** Yujiao Deng, Yuxiu Xie, Meng Wang, Peng Xu, Bajin Wei, Na Li, Ying Wu, Si Yang, Linghui Zhou, Qian Hao, Lijuan Lyu, Dai Zhang, Zhijun Dai

**Affiliations:** ^1^ Department of Breast Surgery, the First Affiliated Hospital, College of Medicine, Zhejiang University, Hangzhou, China; ^2^ Department of Oncology, the 2nd Affiliated Hospital of Xi’an Jiaotong University, Xi’an, China; ^3^ Cancer Center, Union Hospital, Tongji Medical College, Huazhong University of Science and Technology, Wuhan, China

**Keywords:** antihypertensive drugs, colorectal cancer, risk, prognosis, meta-analysis

## Abstract

**Background:** Antihypertensive drugs might play a key role in the risk and poor prognosis of colorectal cancer. However, current epidemiologic evidence remains inconsistent. The aim of this study is to quantify the association between antihypertensive drugs and colorectal cancer.

**Methods:** To identify available studies, we systematically searched electronic databases: PubMed, Web of Science, Embase, Cochrane Library. The risk estimates and their corresponding 95% confidence intervals (CIs) were collected and analyzed by using random-effects models. Heterogeneity test and sensitivity analysis were also performed.

**Results:** Overall, 37 observational studies were included in this analysis (26 studies with cohort design, three studies with nested case-control design, and 8 studies with case-control design). Antihypertensive drugs did not present a significant effect on the risk or overall survival of patients with colorectal cancer [Risk ratio (RR) = 1.00, 95% CI: 0.95–1.04; Hazard ratio (HR) = 0.93, 95% CI: 0.84–1.02]. In the subgroup analysis, diuretics use was significantly associated with a worse overall survival of patients with colorectal cancer (HR = 1.27; 95% CI: 1.14–1.40). However, use of angiotensin-converting enzyme inhibitors/angiotensin II receptor blockers was associated with improved progression-free survival of patients who suffered from colorectal cancer (HR = 0.83; 95% CI: 0.72–0.95).

**Conclusion:** Antihypertensive drug usage did not influence the risk and overall survival of patients with colorectal cancer in general. Further investigation reminded us that diuretics use might reduce the overall survival time in colorectal cancer patients, whereas those who took Angiotensin-converting enzyme inhibitors/angiotensin II receptor blockers had a longer progression-free survival.

## Introduction

Colorectal cancer is the third most commonly cancer in the world, and the second most deadly cancer globally (Sung et al., 2021). Some risk factors, such as genetic, lifestyle (Deng et al., 2021), obesity, and environmental factors, were reported to be associated with colorectal cancer (Dekker et al., 2019). It is estimated that approximately 47% (16.1 million individuals) U.S. residents aged >18 years suffer from hypertension and consequently use antihypertensive agents (Merai et al., 2016). Antihypertensive drugs including angiotensin-converting enzyme inhibitors (ACEI), angiotensin II receptor blockers (ARB), calcium-channel blockers (CCB), beta-blockers (BB) and diuretics are commonly used to lower blood pressure as well as reduce the occurrence and risk of cardiovascular disease (Thomopoulos et al., 2015; Ettehad et al., 2016).

The association between the use of antihypertensive agents and cancer risk have been raised as concerns since 1976. It is reported that the use of Rauwolfia in hypertension patients did not increase the risk of breast cancer (Aromaa et al., 1976). A decade later, a large multicenter screening program consisting of 1,362 cases and 1,250 controls participants, found that long-term usage of Rauwolfia elevated the risk of breast cancer (Stanford et al., 1986). Hallas demonstrated that the long-term use of ACEI increased the risk of colorectal cancer (Hallas et al., 2012), but another study concluded that ARB decreased the risk (Wang et al., 2012). A study of 14,166 patients indicated that long-term diuretics therapy might increase colon cancer-specific mortality (Tenenbaum et al., 2001). A population-based study, with a follow-up time of 6.6 years, supported that pre- or post-diagnostic BB intake was not related with colorectal cancer prognosis (Jansen et al., 2017), but a recent study suggested BB might improve overall survival (OS) (Fiala et al., 2019). In addition, a cohort study from Shanghai proposed that ARB and BB usage were associated with better survival in colorectal cancer patients (Cui et al., 2019). Previous meta-analysis also showed that the usage of ACEI/ARB resulted in a significant improved OS of patients with colorectal cancer (0.90; 95% CI 0.82–0.98; *p* = 0.021), but this conclusion needs to be further verified because only 5 studies were included (Zhou et al., 2020).

From the above, current evidence on the relationship between antihypertensive drugs and the risk and prognosis of colorectal cancer remains inconsistent. And several types of antihypertensive drugs influence the risk and prognosis of colorectal cancer differently. Therefore, we conducted a systematic review and meta-analysis investigating the risk of developing colorectal cancer and prognosis of colorectal cancer among individuals using antihypertensive drugs.

## Materials and Methods

### Data Sources and Search Strategy

The established criteria followed the Preferred Reporting Items for Systematic Reviews and Meta-Analyses (PRISMA) guidelines (Moher et al., 2009). The PRISMA 2009 checklist was shown in Supplementary Table S1. Utilized electronic databases included PubMed, Web of Science, Embase, Cochrane Library. Two authors independently searched for observational data on colorectal cancer from studies published up to April 17, 2020 without any restriction regarding geographical parameters, publication type or language. The search strategy and terms were based on a combination of MeSH terms, keywords, and substance names which were listed in the Supplementary Table S2. In addition, all reference lists of relevant meta-analysis articles and relevant reviews were analyzed to identify additional articles.

### Inclusion and Exclusion Criteria

After excluding duplicate citations, two reviewers independently scanned titles and abstracts to identify initial studies and excluded those which were unrelated. Afterward, full texts of the remaining studies were reviewed for further evaluation. If the two reviewers didn’t agree about inclusion/exclusion of a publication, it was resolved by the adjudicating senior authors (Zhijun Dai), with consensus achieved by discussion.

All studies included fulfilled the following inclusion criteria: (a) studies which were observational study, such as a cohort or case-control design; (b) for risk, patients must not suffer from cancer before using antihypertensive drugs; for prognosis, patients must be diagnosed with colorectal cancer; (c) studies which evaluated the effect of antihypertensive drugs in colorectal cancer risk or prognosis; (d) studies which compared antihypertensive drugs users with not having received any prescription of antihypertensive drugs during the study period; (e) studies which described survival outcomes such as OS, recurrence-free survival (RFS), cancer-specific survival (CSS), progression-free survival (PFS), disease-free survival (DFS); (f) studies that reported effect value such as HR, RR, odds ratio (OR) with their 95% confidence intervals (CIs); (g) the exposure of antihypertensive drugs were clearly defined within the study.

We excluded articles for the following reasons: (a) articles which were meta-analysis, reviews, case reports, experimental laboratory articles, abstracts, animal studies, commentaries, letters; (b) articles which used antihypertensive or other drugs as references.

For studies using the same populations, we included the latest or the longest follow-up study. Two reviewers checked the data of the included studies to prevent duplication.

### Data Extraction

Two reviewers extracted the following information independently: the first author’s name, the geographical location, publication year and population gender, the exposure time and follow-up period, number and characteristics of populations, number of cancer case, number of deaths, cancer sites, study design, types of antihypertensive drugs used, outcome indicator, and effect values with their respective 95% CIs.

### Quality Assessment

Under the guidance of Newcastle-Ottawa Quality Assessment Scale (NOS), the quality of each article was assessed by two reviewers independently. Any disagreements were discussed by the group members until an agreement was reached. As Supplementary Table S3 shown, NOS scores of studies included in this meta-analysis varied from six to eight points and 7–8 scores were considered indicative of high quality.

### Statistical Analysis

Risk estimates with their respective 95% CIs were calculated by using random effect models to estimate the risk and prognosis of colorectal cancer for patients who used antihypertensive drugs, compared with those who did not. The Cochran’s Q test and I^2^ statistic were performed to assess heterogeneity, whereby *p* values <0.1 or I^2^ values >50% represents significant heterogeneity. Subgroup analyses was conducted through stratifying data by geographical locations, cancer sites, study design types, publish date, and NOS score, to elucidate potential sources of heterogeneity. We investigated the publication bias by funnel plots and Egger’s test (Begg and Mazumdar, 1994; Egger et al., 1997). If *p* values was greater than 0.05 in the Egger’s test or it was symmetry in the funnel plot, the publication bias was acceptable. In this study, we conducted sensitivity analysis to assess the effect of each study on the meta-analysis model. All statistical tests were two-sided, and the significance level was 0.05. In addition, all data were analyzed using Stata 12.0 software (Markummitchell, Torrance, CA, United States).

## Results

### The Characteristics of Included Study

**FIGURE 1:**
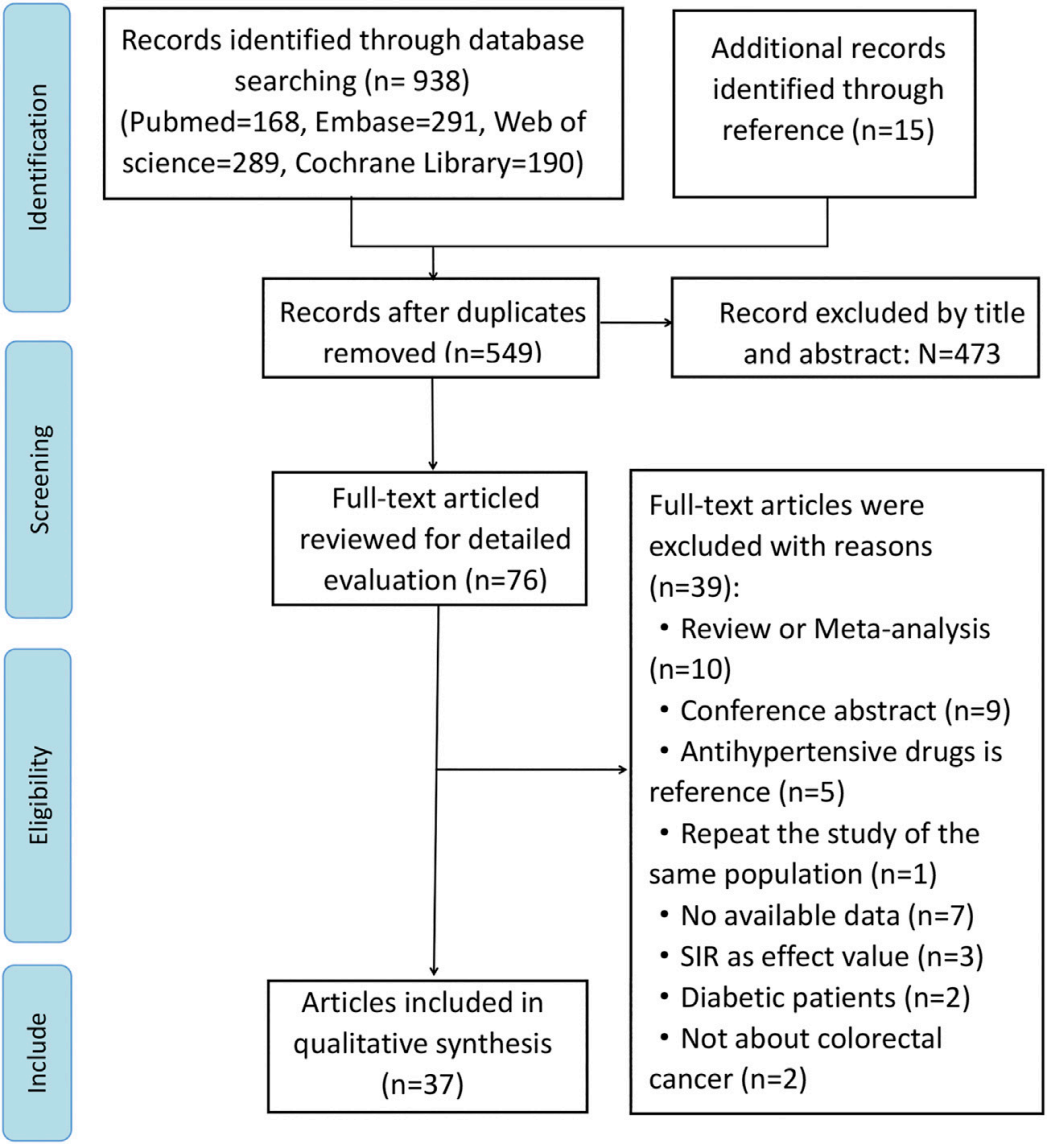
Flow chart of the study procedure.

While 953 articles were initially identified through online searches, 549 articles were retained for analysis after duplicates were removed. Although we searched the above databases without any language restriction, all studies included were published in English. Studies were retrieved by filtering titles and abstracts, and 39 studies were excluded after full text review, and reasons why studies were excluded were listed in Supplementary Table S4. Ultimately, we included 37 studies for our meta-analysis, including 20 publications concerning cancer risk (Pahor et al., 1996; Michels et al., 1998; Rosenberg et al., 1998; Tenenbaum et al., 2001; Beiderbeck-Noll et al., 2003; Boudreau et al., 2008; van der Knaap et al., 2008; Friedman et al., 2011; Hallas et al., 2012; Jansen et al., 2012; Wang et al., 2012; Mansouri et al., 2013; Makar et al., 2014; Chang et al., 2015; Lin et al., 2015; Numbere et al., 2015; Grimaldi-Bensouda et al., 2016; Dierssen-Sotos et al., 2017; Cheung et al., 2020; Brasky et al., 2021), 17 regarding cancer prognosis (Sorensen et al., 2000; Hicks et al., 2013; Holmes et al., 2013; Cardwell et al., 2014; Jansen et al., 2014; Giampieri et al., 2015; Osumi et al., 2015; Morris et al., 2016; Jansen et al., 2017; Weberpals et al., 2017; Sud et al., 2018; Bowles et al., 2019; Cui et al., 2019; Fiala et al., 2019; Mafiana et al., 2019; Ozawa et al., 2019; Ahl et al., 2020). Of these 37 studies, 26 studies used cohort design, three studies used nested case-control design, and 8 studies used case-control design. Our study selection process is illustrated in a flow chart (Figure 1). The total number of participants included in this analysis was 1,117,991, ranging from 107 to 208,635 participants per study. Data was extracted from 13 countries from three continents including North America (*n* = 11 studies), Asia (*n* = 8 studies), and Europe (*n* = 18 studies). Apart from 3 studies (Boudreau et al., 2008; Numbere et al., 2015; Dierssen-Sotos et al., 2017) that did not specify the follow-up time, the follow-up time of the other 34 articles were more than 1 year. Key characteristics of studies included in the meta-analysis are summarized in Table 1.

**TABLE 1:** The characteristics of studies among the association between antihypertensive drugs use and risk or prognosis of colorectal cancer.

Study	Cancer site	Gender	Age	Country	Population	Cancer case	Diagnosis period	Follow-up period	Medicine	Study design	NOS
Risk											
Beiderbeck-Noll et al. (2003)	Colon-Rectum	MIX	≥71	Netherlands	3204	59	January 1, 1991 and January 1, 1999	16, 640 person-years (mean 5.2 years)	CCB	cohort	8
Boudreau et al. (2008)	Colorectum	MIX	70 (mean)	USA	665	357	January 1, 2000, and December 31, 2003	NA	ACEI/CCB/Diuretics	case–control	6
Brasky, et al. (2021)	Colorectum	FEMALE	50–79	USA	142,812	2,185	1993–2020	10 years	ACEI/ARB	cohort	8
Chang, et al. (2015)	Colon	MIX	53.5 (mean)	China	24,238	68	January 1, 2000 and December 31, 2011	12 years	BB	cohort	7
Cheung et al. (2020)	Colorectum	MIX	60.6 (52.3–71.9)	China	187,897	854	2005–2017	3 years	ACEI/ARB	cohort	8
Dierssen Sotos et al. (2017)	Colorectum	MIX	65.1 (20–85)	Spain	6077	NA	January 1st, 2007 and March 31st, 2012	NA	ACEI/ARB	case–control	7
Friedman et al. (2011)	Colon	MIX	NA	USA	NA	1971	August 1994 through February 2008	>2 years	BB(Atenolol/metoprolol/propanolol)	case–control	6
Grimaldi-Bensouda et al. (2016)	Colon	MIX	61.3 (mean)	UK	150750	14588	1996–2009	at least 2 years	CCB	cohort	8
Hallas et al. (2012)	Colorectum	MIX	69.4 (mean)	Denmark	149, 417	30683	2000–2005	7.8 years	ACEI/ARB	case–control	7
Jansen et al. (2012)	Colorectum	MIX	67.7 (±10.5)	Germany	3470	762	2003 and 2007	>2 years	BB	case–control	8
Lin et al. (2015)	Colon	MIX	62.8 (±12.8)	China	13,542	70	2000 and 2010	4.93 years, 5.17 years	BB	cohort	8
Makar et al. (2014)	Colorectum	MIX	69.8 (±9.1)	UK	2847	4316	1987–2002	>1year	ACEI/ARB/BB/CCB/Diuretics	nest case–control	7
Mansouri et al. (2013)	Colorectum	MIX	50–74	UK	4188	371	April 2009 to March 2011	>1year	ACEI	cohort	7
Michels et al. (1998)	Colorectum	FEMALE	NA	USA	18,635	6	1988–1994	6 years, 107,256 person-years	CCB	cohort	8
Numbere et al. (2015)	Colorectum	MIX	≥18	UK	208,635	18968	January 1, 1987 and December 31, 2012	NA	BB/CCB	case–control	8
Pahor et al. (1996)	Colon-Rectum	MIX	≥71	USA	5,052	88	1988–1992	3.27 years, 18,774 person-years	CCB	cohort	7
Rosenberg et al. (1998)	Colon-Rectum	MIX	46–69	USA	9513	302	1976 to 1996	>1year	ACEI/BB/CCB/	case–control	7
Tenenbaum et al. (2001)	Colon	MIX	61.8 ± 6.2	Israel	1023	23	February 1, 1990, and October 30, 1992	4–7 years (mean 5.6 ± 0.8 years)	Diuretics	cohort	8
van der Knaap et al. (2008)	Colorectum	MIX	70.4 ± 9.7	Netherlands	730	129	July 1989 and July 1993	9.6 years	ACEI/ARB	cohort	7
Wang et al. (2012)	Colon	MIX	62 ± 13	China	42921	187	January 1997–December 2009	4.8 ± 2.4 years	ARB	cohort	8
Overall Survival											
Ahl et al. (2020)	Rectal	MIX	72.2 ± 9.3	Sweden	11966	776	January 1, 2007–October 31, 2016	1 year	BB	cohort	6
Cardwell et al. (2014)	Colorectum	MIX	NA	UK	4762	2444	1998–2006	6 years	ACEI/ARB	nest case–control	7
Cui et al. (2019)	Colorectum	MIX	40–74	China	890	383	hanghai Women’s Health Study (1996–2000), Shanghai Men’s Health Study (2002–2006)	4 years	ACEI/ARB/BB/CCB/Diuretics	cohort	8
Fiala et al. (2019)	Colorectum	MIX	63.2 (28.0–86.1)	Czech Republic	514	345	2005–2019	519 days	ACEI/ARB/BB/CCB	cohort	7
Giampieri et al. (2015)	Colorectum	MIX	61 (37–85)	Italy	235	29	2010 and 2013	41.3 vs. 25.7 months	BB	cohort	6
Hicks et al. (2013)	Colorectum	MIX	NA	UK	4794	1559	1998 and 2007	6.2 years (range 1–13.9)	BB	nest case–control	8
Holmes et al. (2013)	Colorectum	MIX	70 ± 13	Canada	3967	3824	2004 and 2008	>1year	ACEI/ARB/BB/CCB/Diuretics	cohort	7
Jansen et al. (2014)	Colorectum	MIX	70.2 ± 9.1	Germany	1975	187	2003 and 2007	5.0 years	BB	cohort	7
Jansen et al. (2017)	Colon-Rectum	MIX	73 ± 9	Germany	8100	919	1998 and 2011	6.6 years, 4639 person-years	BB	cohort	8
Mafiana et al. (2019)	Colorectum	MIX	55 ± 15.15	Arab	301	NA	2006–2014		ACEI/ARB	cohort	6
Morris et al. (2016)	Rectal	MIX	61.1 ± 11.5	USA	261	74	January 1, 1999 and July 1, 2012	5.3 years	ACEI/ARB	cohort	7
Osumi et al. (2015)	Colorectum	MIX	61.5 (38–75)	Japan	181	104	June, 2007 and September, 2010	2.2 years (26.7 months)	ARB	cohort	7
Sorensen et al. (2000)	Colon	MIX	64.9 ± 13.1	Denmark	27788	82	January 1, 1989 and December 31, 1995	3.2 years, 73,193 person-years	CCB	cohort	8
Weberpals et al. (2017)	Colorectum	MIX	71.2 ± 8.7	Netherlands	3572	1553	April 1, 1998 and December 31, 2011	6.3 years	BB	cohort	7
Progression-free survival											
Bowles et al. (2019)	Colon	MIX	69.9 ± 11.6	USA	2039	760	1995–2014	4.9 years	ACEI/ARB/BB/CCB/Diuretics	cohort	8
Fiala et al. (2019)	Colorectum	MIX	63.2 (28.0–86.1)	Czech Republic	514	296	2005–2019	519 days	ACEI/ARB/BB/CCB	cohort	7
Giampieri et al. (2015)	Colorectum	MIX	61 (37–85)	Italy	235	29	2010 and 2013	8.36 vs. 7.13 months	BB	cohort	6
Jansen et al. (2014)	Colorectum	MIX	70.2 ± 9.1	Germany	1975	91	2003 and 2007	5.0 years	BB	cohort	7
Morris et al. (2016)	Rectal	MIX	61.1 ± 11.5	USA	261	74	January 1, 1999 and July 1, 2012	5.3 years	ACEI/ARB	cohort	7
Osumi et al. (2015)	Colorectum	MIX	61.5 (38–75)	Japan	181	104	June, 2007 and September, 2010	2.2 years (26.7 months)	ARB	cohort	7
Ozawa et al. (2019)	Colorectum	MIX	NA	USA	461	94	2009–2014	57 mouths	ACEI/ARB	cohort	7
Sud et al. (2018)	Colorectum	MIX	66.9 (42.9–88.1)	Canada	572	NA	NA	NA	BB	cohort	6

Abbreviations: MIX: male and female; NA: not available; NOS: Newcastle-Ottawa quality assessment scale; ACEI: angiotensin-converting enzyme inhibitors; ARB: angiotensin II receptor blockers; CCB: calcium-channel blockers; BB: beta-blockers.

### Antihypertensive Drugs and Risk of Colorectal Cancer

**FIGURE 2:**
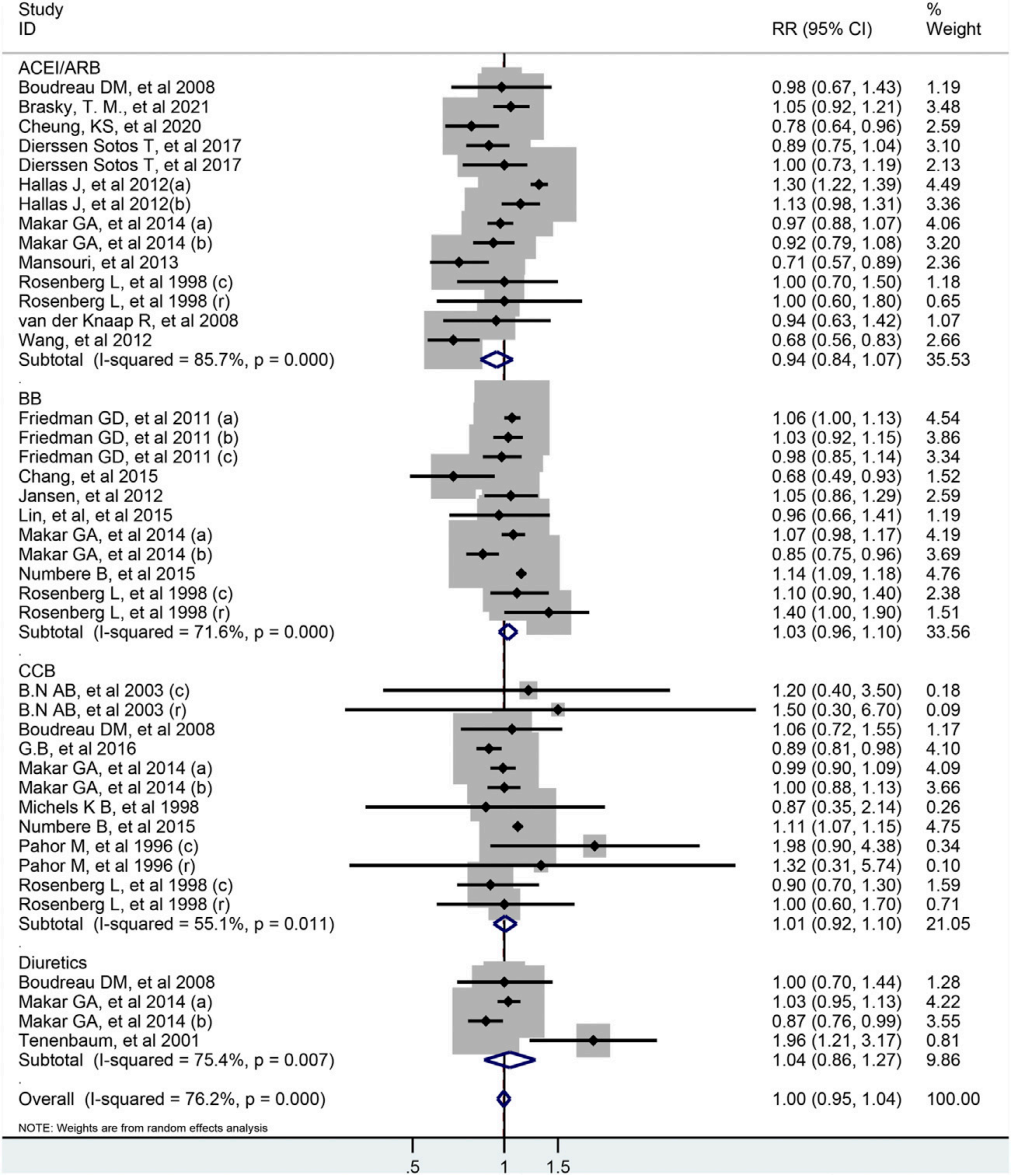
Forest plot of studies among the risk of colorectal cancer with all antihypertensive drugs.

As shown in Figure 2, all antihypertensive drugs were not associated with colorectal cancer risk (RR = 1.00; 95% CI: 0.95–1.04).

**FIGURE 3:**
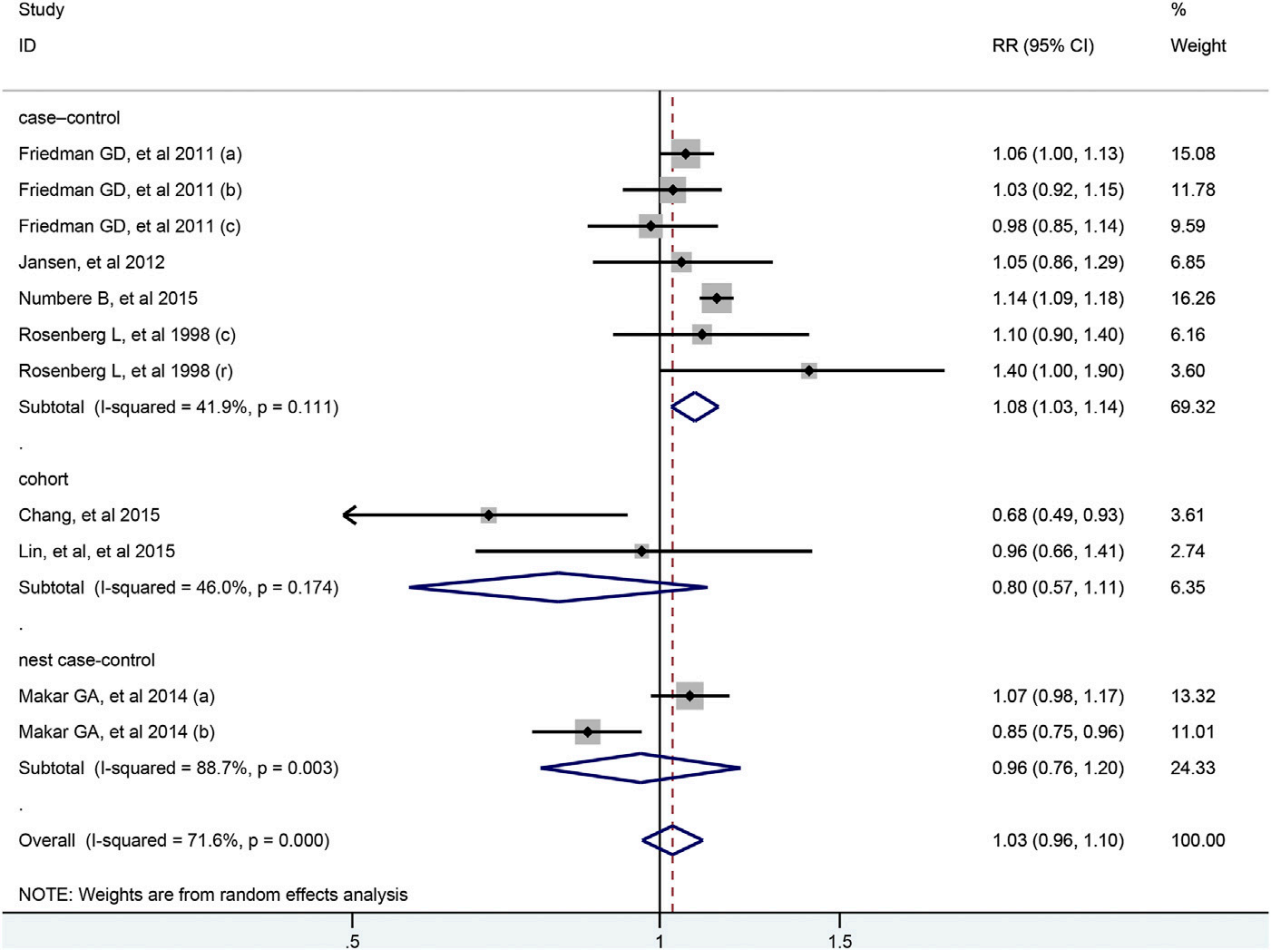
Forest plot of studies among the risk of colorectal cancer with beta-blockers.

There were 11 studies evaluated the link between the risk of colorectal cancer and BB, including two nested case-control studies, two cohort studies and seven case-control studies. As illustrated in Figure 3, no association was shown between BB and the risk of colorectal cancer (RR = 1.03; 95% CI: 0.96–1.10). And the results were robust when the subgroup analysis was stratified by cancer sites or geographical districts. However, the usage of BB significantly increased the risk of colorectal cancer in seven case-control studies (RR = 1.08; 95% CI: 1.03–1.14) and in the high-quality study with NOS score of 8 (RR = 1.13; 95% CI: 1.09–1.18), but not in two cohort studies (RR = 0.80; 95% CI: 0.57–1.11) and two nest-cohort studies (RR = 0.96; 95% CI: 0.76–1.20) (Table 2). In the Supplementary Figure S1, the association between colorectal cancer risk and duration of BB exposure was represented by forest plot. Only one study reported that the risk of colon cancer decreased markedly when the patients used BB for longer than 1,000 days (Chang et al., 2015).

**TABLE 2:** The association between antihypertensive drugs use and risk of colorectal cancer.

Comparison	BB vs. non				CCB vs. non				ACEI/ARB vs. non			
Category	N	RR (95%CI)	*I* ^*2*^ (%)	*P*	N	RR (95%CI)	*I* ^*2*^ (%)	*P*	N	RR (95%CI)	*I* ^*2*^ (%)	*P*
Colorectum	11	1.03 (0.96–1.10)	71.6	0.000	12	1.01 (0.92–1.10)	55.1	0.011	14	0.94 (0.84–1.07)	85.7	0.000
Cancer site												
Colon	6	1.01 (0.94–1.10)	38.8	0.147	4	0.94 (0.78–1.14)	27.5	0.247	2	0.80 (0.55–1.15)	67.8	0.078
Rectal	1	1.40 (1.02–1.93)	NA	NA	3	1.07 (0.67–1.70)	0.0	0.850	1	1.00 (0.58–1.73)	NA	NA
Study design												
Case-control	7	**1.08 (1.03–1.14)**	41.9	0.111	4	**1.10 (1.06–1.15)**	0.0	0.600	7	1.06 (0.91–1.23)	74.8	0.001
Cohort	2	0.80 (0.57–1.11)	46	0.174	6	**0.90 (0.82–0.99)**	0.0	0.439	5	0.82 (0.67–1.00)	77.2	0.002
NCC	2	0.96 (0.76–1.20)	88.7	0.003	2	0.99 (0.92–1.07)	0.0	0.900	2	0.96 (0.88–1.04)	0.0	0.574
Geographic area												
North America	5	1.05 (1.00–1.11)	6.8	0.368	6	1.01 (0.83–1.24)	0.0	0.607	4	1.04 (0.92–1.17)	0.0	0.983
Europe	4	1.03 (0.91–1.16)	85.2	0.0	6	1.00 (0.90–1.11)	75.9	0.001	8	0.98 (0.85–1.14)	88.2	0.000
Asia	2	0.80 (0.57–1.11)	46	0.174	NA	NA	NA	NA	2	**0.73 (0.63–0.84)**	0.0	0.341
Publish date												
1995–2000	2	1.21 (0.96–1.52)	32.1	0.225	5	1.00 (0.78–1.27)	0.0	0.472	2	1.00 (0.73–1.37)	0.0	1.000
2000–2010	NA	NA	NA	NA	3	1.09 (0.77–1.55)	0.0	0.899	2	0.96 (0.73–1.27)	0.0	0.883
2010–2020	9	1.01 (0.94–1.09)	75.5	0.000	4	1.00 (0.90–1.11)	85.4	0.000	10	0.94 (0.81–1.08)	90.0	0.000
NOS score												
6	3	1.04 (0.99–1.10)	0.0	0.604	1	1.06 (0.72–1.56)	NA	NA	1	0.98 (0.67–1.43)	NA	NA
7	5	0.99 (0.83–1.18)	79.4	0.001	6	0.99 (0.93–1.07)	0.0	0.628	10	0.98 (0.86–1.13)	84.9	0.000
8	3	**1.13 (1.09–1.18)**	0.0	0.521	5	1.00 (0.83–1.21)	77.4	0.001	3	0.83 (0.63–1.09)	86.1	0.001

Abbreviations: ACEI: angiotensin-converting enzyme inhibitors; ARB: angiotensin II receptor blockers; CCB: calcium-channel blockers; BB: beta-blockers; RR: relative risk; CI: confidence intervals; N: number of studies, NCC: nest case-control; NA, not available.

Bold indicates values which are statistically significant.

**FIGURE 4:**
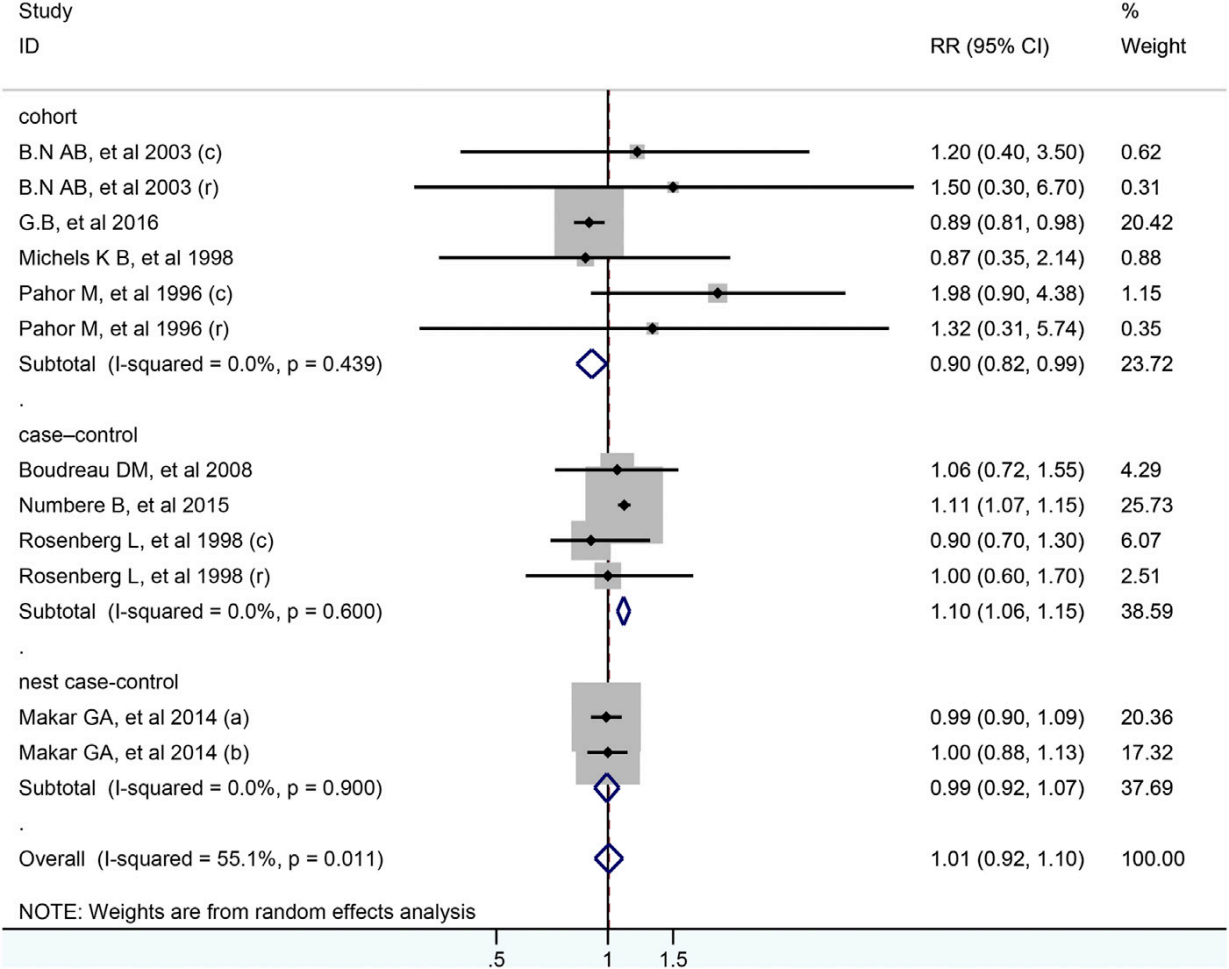
Forest plot of studies among the risk of colorectal cancer with calcium-channel blockers.

In total, 12 studies including 399,301 participants were analyzed for the link between CCB use and colorectal cancer risk. After pooled analysis, no significant association was observed between CCB and risk of colorectal cancer (RR = 1.01; 95% CI: 0.92–1.10, Figure 4). Meta-analysis of four case-control studies revealed that the pooled RR was 1.10 (95% CI: 1.06–1.15, I^2^ =0.0%), while it was 0.90 (95% CI: 0.82–0.99) for six cohort studies. As for different cancer sites, geographical districts subgroup, publish date and NOS score, no significant association was observed (Table 2). The detailed duration exposure data to CCB and the risk of colorectal cancer were shown in Supplementary Figure S2.

**FIGURE 5:**
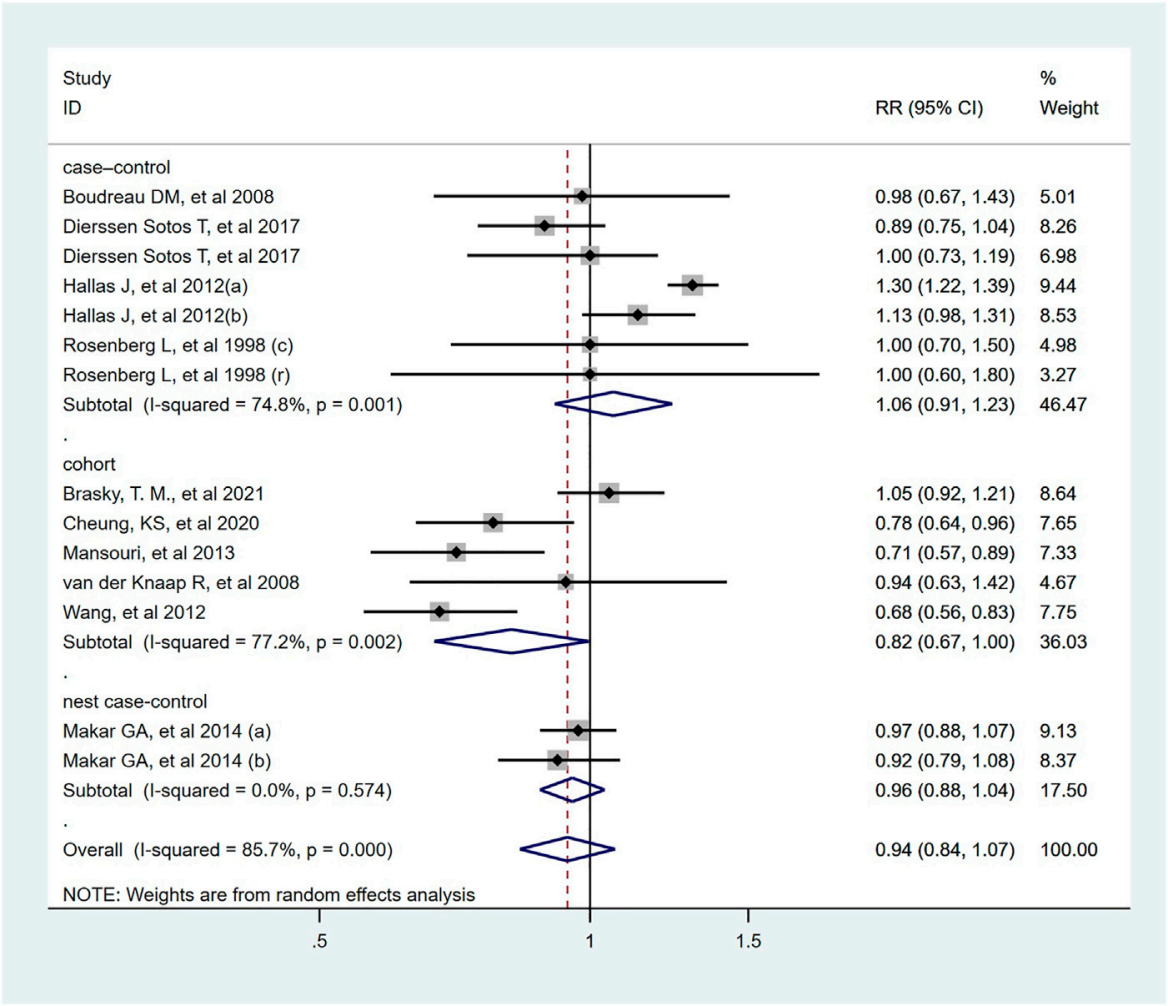
Forest plot of studies among the risk of colorectal cancer with angiotensin-converting enzyme inhibitors/angiotensin II receptor blockers.

Fourteen studies indicated that the usage of ACEI/ARB was not significantly associated with risk of colorectal cancer (RR = 0.94; 95% CI: 0.84–1.07, Figure 5). In subgroup analysis, these results were robust, and were consistent irrespective of study design type, cancer site, publish date or NOS score. However, two cohort study from Asian population found that used ACEI/ARB was related with reduced risk of colorectal cancer (RR = 0.73; 95% CI: 0.63–0.84), but there was no significant association in Europe and North America. In addition, the pooled RR was 0.97 (95% CI: 0.75–1.25) for ACEI users, and 0.92 (95% CI: 0.67–1.27) for ARB users. The detailed duration exposure data to ACEI/ARB and the risk of colorectal cancer were shown in Supplementary Figure S3.

**FIGURE 6:**
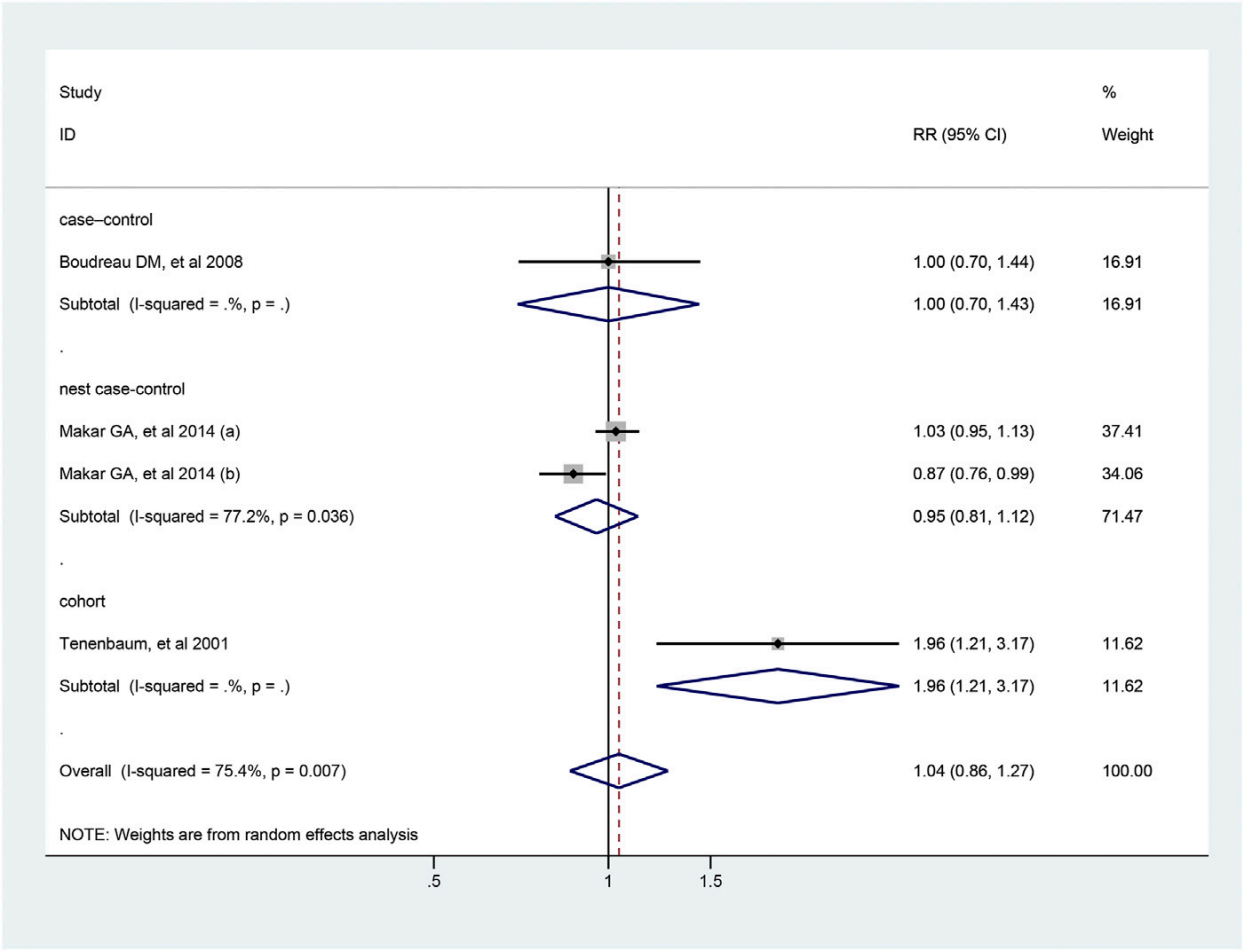
Forest plot of studies among the risk of colorectal cancer with diuretics.

Four studies reported the association between the risk of colorectal cancer and usage of diuretics, and pooled analysis showed a RR value of 1.04 (95% CI: 0.86–1.27, Figure 6). Due to the limited number of studies included, further analysis could not be conducted. The detailed duration exposure data to diuretics and the risk of colorectal cancer were shown in Supplementary Figure S4.

### Antihypertensive Drugs and Overall Survival of Colorectal Cancer

**FIGURE 7:**
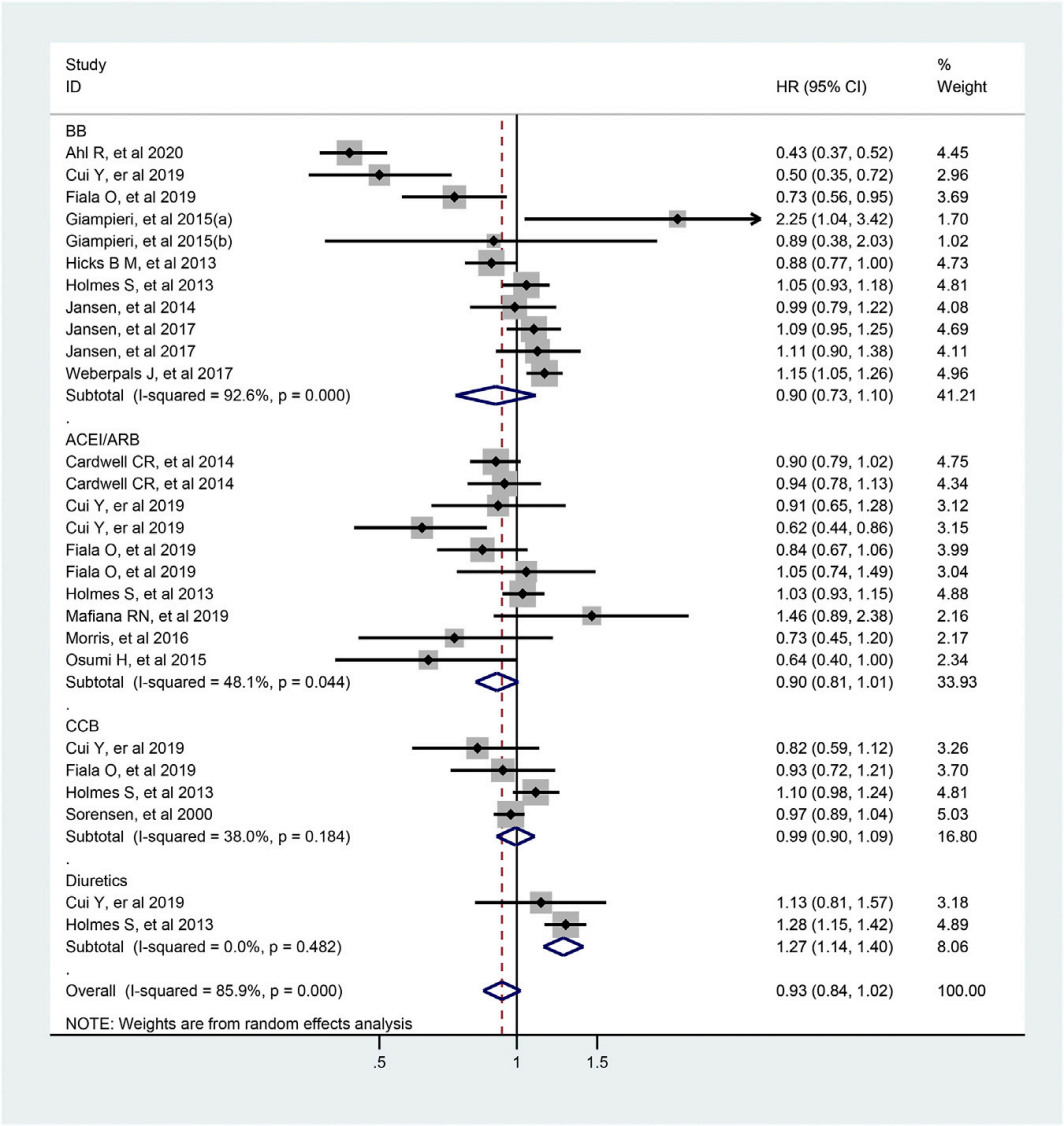
Forest plot of studies among the OS of colorectal cancer patients with antihypertensive drugs.

As shown in Figure 7, 27 articles focused on association between the usage of antihypertensive drugs and OS of patients with colorectal cancer. Totally, antihypertensive drugs use was not associated with improved OS of patients with colorectal cancer (HR = 0.93; 95% CI: 0.84–1.02). As for the subtype of antihypertensive drugs, usage of diuretics was significantly associated with a worse OS of colorectal cancer patients (HR = 1.27; 95% CI: 1.14–1.40). However, similar effect was not observed in those who used ACEI/ARB, BB or CCB (ACEI/ARB: HR = 0.90, 95% CI: 0.81–1.01; BB: HR = 0.90, 95% CI: 0.93–1.10; CCB: HR = 0.99, 95% CI 0.90–1.09; respectively).

### Antihypertensive Drugs and Progression Free Survival of Colorectal Cancer

**FIGURE 8:**
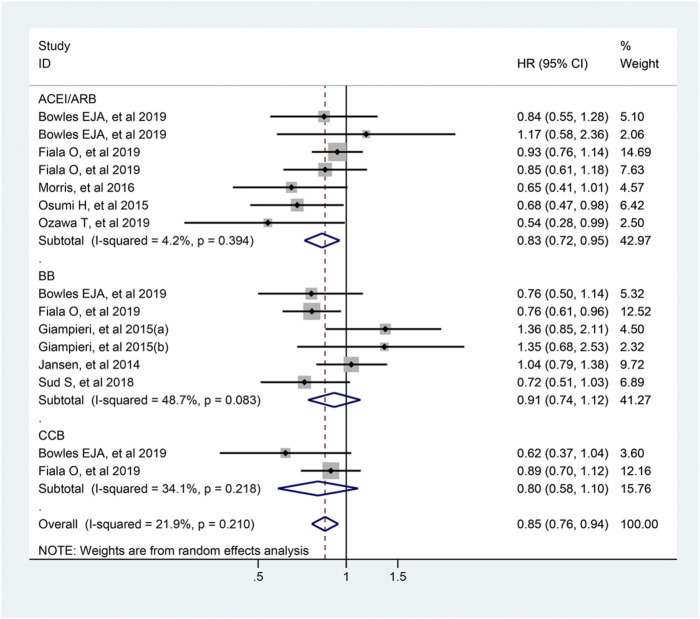
Forest plot of studies among the PFS of colorectal cancer patients with antihypertensive drugs.

The pooled estimates of 15 studies which included 3,072 participants, demonstrated that the usage of antihypertensive drugs was related to longer PFS of colorectal cancer patients (HR = 0.85; 95% CI: 0.76–0.94, Figure 8). In the subgroup analysis, ACEI/ARB users were associated with a better PFS compared with non-users (HR = 0.83; 95% CI: 0.72–0.95), but not for BB and CCB users.

### Publication Bias

We performed the Begg’s funnel plot and Egger’s test to investigate publication bias (Supplementary Figures S5, S6). No apparent indication of publication bias between colorectal cancer risk and BB/CCB/diuretics users, except for ACEI/ARB users (Egger’s: *p* = 0.018; Begg’s tests: *p* = 0.827). For prognosis of colorectal cancer patients, both Egger’s (OS: *p* = 0.159, PFS: *p* = 0.657) and Begg’s tests (OS: *p* = 0.243, PFS: *p* = 0.499) showed no significant publication bias.

### Sensitivity Analysis

Each study was individually eliminated to access the effect of individual studies on the results (Supplementary Figures S7, S8). In the analysis of colorectal cancer risk with CCB users, the pooled RR was statistically significant after deletion of one article (RR = 1.07; 95% CI: 1.03–1.12) (Grimaldi-Bensouda et al., 2016). However, the other results were not influenced significantly when we removed each article.

## Discussion

This meta-analysis included 37 observational studies involving a large number of participants to quantify the association between usage of antihypertensive drugs and risk as well as prognosis of colorectal cancer. Overall, the usage of antihypertensive drugs was not associated with the risk or OS of colorectal cancer, which is accordant with previous published researches. In 2011, a network analysis rejected the hypothesis that the usage of antihypertensive agents was linked with a relative increase in the occurrence of cancer or cancer-specific death (Bangalore et al., 2011). After that, Ioannidis and colleagues conducted an umbrella review of 74 meta-analysis studies and stated that no medication was proven to increase the risk of cancer (Ioannidis et al., 2014).

Interestingly, our further analysis identified that antihypertensive drugs might improve PFS of colorectal cancer patients, especially for ACEI/ARB users, which is similar to the recent published meta-analysis, which suggested that ACEI/ARB improved OS of colorectal cancer patients (Zhou et al., 2020). Actually, in our pooled analysis with more included studies, the usage of ACEI/ARB was not associated with risk and OS of colorectal cancer, which is consistent with the study of Sipahi et al., which concluded that the usage of ACEI did not affect the risk or survival of patients with cancer through a meta-analysis of 10 RCTs and 59,004 patients (Sipahi et al., 2010). Previous studies indicated that ACEI/ARB affected cancer prognosis by suppressing cancer proliferation and angiogenesis, and promoting cell apoptosis (Ager et al., 2008; George et al., 2010). A review summarized ACEI/ARB presented a potential effect in colorectal cancer by inhibiting vascular endothelial growth factor and insulin-like growth factor 1, and the usage of ACEI suppressed the development and metastasis of colorectal cancer (Asgharzadeh et al., 2018). In addition, a potential indirect antitumor mechanism of ACEI/ARB was found to enhance the delivery of antitumor drugs into tumor tissues (Maeda et al., 2013). Consistent with our results, McMenamin and colleagues conducted a systematic review and proposed that ACEI or ARB use might improve outcome of colorectal cancer patients (Mc Menamin Ú et al., 2012). Besides, usage of ACEI/ARB significantly increased the rate of pathological complete regression after neoadjuvant treatment in rectal cancer (Morris et al., 2016). Therefore, the ACEI/ARB use for colorectal cancer patients with hypertension might be suggested.

Heterogeneity, though unavoidable, cannot be ignored. In all meta-analysis, the cause of the heterogeneity should be searched for and analyzed. After conducting subgroup analyses by geographical locations, cancer sites, study design, publish date, and NOS score, the heterogeneity was reduced significantly. First, given that most colorectal cancer and hypertensive populations are old people, age is an essential risk factor for colorectal cancer. One study observed a statistically significant protective effect for ACEI users from colorectal cancer, which was restricted to the under 65 years old group (Dierssen-Sotos et al., 2017). In our meta-analysis, the average age of the population in most studies was over 65 and analysis in each study was adjusted on the basis of age, only few studies did not indicate the age distribution or include all people over 18 years old. Therefore, few different distribution of age might cause some heterogeneity. Second, subgroup analyses suggested that different research design can partially explain heterogeneity across the study. For example, the heterogeneity was reduced obviously after subgroup analyses by research design was conducted. In the subgroup of case-control study, the use of CCB was associated with an increased risk of colorectal cancer. However, in the subgroup of cohort study, CCB might play an anti-cancer effect. These two opposite conclusions indicated that the association between CCB use and the risk of colorectal cancer were still controversial, and more research was needed to verify the true connection between them. Third, ethnic variation may explain bias and heterogeneity. According to the Taiwan National Health Insurance and the Hong Kong Hospital Authority research database, two cohort studies found that usage of ACEI/ARB was related with a decrease in the colorectal cancer risk. However, there were not positive association for the included studies from North American and Europe. Several studies also provided evidence that ethnic variation influenced the efficacy of antihypertensive drugs (Gupta et al., 2010; Ogedegbe et al., 2015). In fact, the major of included studies in our analysis were conducted in Europe and North America. To an extent, these cohort studies may provide accurate and consistent baseline data due to their similar geographical conditions. Further large well-conducted prospective studies from Asia are required to confirm our results. In the end, given the very heterogenous nature of studies included (in terms of study years, exposure assessment from different databases, outcomes and covariates assessment and analytical strategies from different studies), may partly account for heterogeneity in our study. Furthermore, we conducted subgroup analyses stratifying on NOS scores, and the heterogeneity was decreased significantly.

Antihypertensive drugs might promote or interfere with tumor cell proliferation, migration and apoptosis, as well as angiogenesis (Grossman et al., 2001; Greene and Amaral, 2002; Kanehira et al., 2005; Tang et al., 2013; Granados et al., 2020). CCB was found to inhibit the spreading of neoplastic cells by regulating cell proliferation and calcium influx (Grossman et al., 2001). Additionally, it could enhance the anti-tumor effects of chemotherapy drugs, and participate in the regulation of cell differentiation, death, and susceptibility to MAPK inhibitors *in vitro* and *in vivo* (Granados et al., 2020) The expression of beta-adrenergic receptors were at high level in a large number of cancer cells, which could be activated and promote the process of tumor progression, including anti-apoptosis, proliferation, angiogenesis, invasion, and metastasis (Tang et al., 2013). ACEI/ARB has been proven to inhibit angiogenesis, tumor proliferation and metastasis (Greene and Amaral, 2002; George et al., 2010). However, some animal experiments supported that ACEI/ARB promoted tumor growth by increasing the expression of vascular endothelial growth factor (VEGF), reducing the level of platelet reactive protein 1 in the tissue and transforming growth factor-beta-dependent cell growth (Kanehira et al., 2005; Clere et al., 2010). Currently, the mechanism underlying the possible causal links between antihypertensive drugs and cancer risk are controversial and needs further investigation.

Our investigation was limited by several factors. Firstly, despite using the random effect model, our results should be treated with caution due to significant heterogeneity and limited data. Though most studies we included had adjusted for confounding factors, such as age, BMI, sex, race, outcome value, social background et al., the heterogeneity still exist. Secondly, potential deviations, such as recall deviations, detection deviations, selection deviation and confounding factors, have to be considered in observational studies. Thirdly, mild publication bias was detected when analyzing studies of risk. In addition, our sensitivity analysis showed that the association between colorectal cancer risk and CCB was unstable and controversial. Finally, due to the lack of relevant data, we cannot conduct a dose-response association between duration of antihypertensive drugs exposure and the development of colorectal cancer. It should be noted that all studies included in this analysis included participants who were middle-aged and older, so the results cannot be applied to the general population or children.

In summary, there was no sufficient evidence to prove that antihypertensive drug usage had an impact on the risk and OS of colorectal cancer. Our findings indicated that ARB/ACEI use might improve the PFS of colorectal cancer. More well-designed prospective studies are needed to support our findings.

## Data Availability

The original contributions presented in the study are included in the article/Supplementary Material, further inquiries can be directed to the corresponding author.

## Abbreviations

ACEI, angiotensin-converting enzyme inhibitors; ARB, angiotensin II receptor blockers; BB, beta-blockers; CC, case-control; CCB, calcium-channel blockers; CI, confidence interval; HR, hazard ratio; MIX, male and female; NA, not available; NCC, nest case-control; OR, odds ratio; OS, overall survival; PFS, progression-free survival; RR, risk ratio.

## References

[B1] Ager E. I. Neo J. Christophi C. (2008). The Renin-Angiotensin System and Malignancy. Carcinogenesis 29 (9), 1675–1684. 10.1093/carcin/bgn171 18632755

[B2] Ahl R. Matthiessen P. Fang X. Cao Y. Sjolin G. Lindgren R. (2020). β-Blockade in Rectal Cancer Surgery: A Simple Measure of Improving Outcomes. Ann. Surg. 271 (1), 140–146. 10.1097/SLA.0000000000002970 30048321

[B3] Aromaa A. Hakama M. Hakulinen T. Saxén E. Teppo L. Idä lan-Heikkilä J. (1976). Breast Cancer and Use of Rauwolfia and Other Antihypertensive Agents in Hypertensive Patients: a Nationwide Case-Control Study in Finland. Int. J. Cancer 18 (6), 727–738. 10.1002/ijc.2910180603 992904

[B4] Asgharzadeh F. Hassanian S. M. Ferns G. A. Khazaei M. Hasanzadeh M. (2018). The Therapeutic Potential of Angiotensin-Converting Enzyme and Angiotensin Receptor Inhibitors in the Treatment of Colorectal Cancer: Rational Strategies and Recent Progress. Curr. Pharm. Des. 24 (39), 4652–4658. 10.2174/1381612825666190111145140 30636592

[B5] Bangalore S. Kumar S. Kjeldsen S. E. Makani H. Grossman E. Wetterslev J. (2011). Antihypertensive Drugs and Risk of Cancer: Network Meta-Analyses and Trial Sequential Analyses of 324,168 Participants from Randomised Trials. Lancet Oncol. 12 (1), 65–82. 10.1016/s1470-2045(10)70260-6 21123111

[B6] Begg C. B. Mazumdar M. (1994). Operating Characteristics of a Rank Correlation Test for Publication Bias. Biometrics 50 (4), 1088–1101. 10.2307/2533446 7786990

[B7] Beiderbeck-Noll A. B. Sturkenboom M. C. van der Linden P. D. Herings R. M. Hofman A. Coebergh J. W. (2003). Verapamil Is Associated with an Increased Risk of Cancer in the Elderly: the Rotterdam Study. Eur. J. Cancer 39 (1), 98–105. 10.1016/s0959-8049(02)00157-0 12504665

[B8] Boudreau D. M. Koehler E. Rulyak S. J. Haneuse S. Harrison R. Mandelson M. T. (2008). Cardiovascular Medication Use and Risk for Colorectal Cancer. Cancer Epidemiol. Biomarkers Prev. 17 (11), 3076–3080. 10.1158/1055-9965.Epi-08-0095 18957524 PMC2675612

[B9] Bowles E. J. A. Yu O. Ziebell R. Chen L. Boudreau D. M. Ritzwoller D. P. (2019). Cardiovascular Medication Use and Risks of colon Cancer Recurrences and Additional Cancer Events: a Cohort Study. BMC Cancer 19 (1), 270. 10.1186/s12885-019-5493-8 30917783 PMC6437861

[B10] Brasky T. M. Flores K. F. Larson J. C. Newton A. M. Shadyab A. H. Watanabe J. H. (2021). Associations of Angiotensin-Converting Enzyme Inhibitor or Angiotensin Receptor Blocker Use with Colorectal Cancer Risk in the Women's Health Initiative. Cancer Epidemiol. Biomarkers Prev. 30 (5), 1029–1032. 10.1158/1055-9965.EPI-20-1401 33627382 PMC8102324

[B11] Cardwell C. R. Mc Menamin U. C. Hicks B. M. Hughes C. Cantwell M. M. Murray L. J. (2014). Drugs Affecting the Renin-Angiotensin System and Survival from Cancer: a Population Based Study of Breast, Colorectal and Prostate Cancer Patient Cohorts. BMC Med. 12, 28. 10.1186/1741-7015-12-28 24521426 PMC3926686

[B12] Chang P. Y. Huang W. Y. Lin C. L. Huang T. C. Wu Y. Y. Chen J. H. (2015). Propranolol Reduces Cancer Risk: A Population-Based Cohort Study. Medicine (Baltimore) 94 (27), e1097. 10.1097/md.0000000000001097 26166098 PMC4504645

[B13] Cheung K. S. Chan E. W. Seto W. K. Wong I. C. K. Leung W. K. (2020). ACE (Angiotensin-Converting Enzyme) Inhibitors/Angiotensin Receptor Blockers Are Associated with Lower Colorectal Cancer Risk: A Territory-wide Study with Propensity Score Analysis. Hypertension 76 (3), 968–975. 10.1161/HYPERTENSIONAHA.120.15317 32623923

[B14] Clere N. Corre I. Faure S. Guihot A. L. Vessières E. Chalopin M. (2010). Deficiency or Blockade of Angiotensin II Type 2 Receptor Delays Tumorigenesis by Inhibiting Malignant Cell Proliferation and Angiogenesis. Int. J. Cancer 127 (10), 2279–2291. 10.1002/ijc.25234 20143398

[B15] Cui Y. Wen W. Zheng T. Li H. Gao Y. T. Cai H. (2019). Use of Antihypertensive Medications and Survival Rates for Breast, Colorectal, Lung, or Stomach Cancer. Am. J. Epidemiol. 188 (8), 1512–1528. 10.1093/aje/kwz106 31062847 PMC6670048

[B16] Dekker E. Tanis P. J. Vleugels J. L. A. Kasi P. M. Wallace M. B. (2019). Colorectal Cancer. The Lancet 394 (10207), 1467–1480. 10.1016/s0140-6736(19)32319-0 31631858

[B17] Deng Y. Wei B. Zhai Z. Zheng Y. Yao J. Wang S. (2021). Dietary Risk-Related Colorectal Cancer Burden: Estimates from 1990 to 2019. Front. Nutr. 8, 690663. 10.3389/fnut.2021.690663 34504859 PMC8421520

[B18] Dierssen-Sotos T. Gomez-Acebo I. Palazuelos C. Rodriguez-Moranta F. Perez-Gomez B. Fernandez Vazquez J. P. (2017). Relationship between Drugs Affecting the Renin-Angiotensin System and Colorectal Cancer: The MCC-Spain Study. Prev. Med. 99, 178–184. 10.1016/j.ypmed.2017.01.011 28131779

[B19] Egger M. Davey Smith G. Schneider M. Minder C. (1997). Bias in Meta-Analysis Detected by a Simple, Graphical Test. Bmj 315 (7109), 629–634. 10.1136/bmj.315.7109.629 9310563 PMC2127453

[B20] Ettehad D. Emdin C. A. Kiran A. Anderson S. G. Callender T. Emberson J. (2016). Blood Pressure Lowering for Prevention of Cardiovascular Disease and Death: a Systematic Review and Meta-Analysis. Lancet 387 (10022), 957–967. 10.1016/s0140-6736(15)01225-8 26724178

[B21] Fiala O. Ostasov P. Sorejs O. Liska V. Buchler T. Poprach A. (2019). Incidental Use of Beta-Blockers Is Associated with Outcome of Metastatic Colorectal Cancer Patients Treated with Bevacizumab-Based Therapy: A Single-Institution Retrospective Analysis of 514 Patients. Cancers (Basel) 11 (12), 1856. 10.3390/cancers11121856 PMC6966537 31769417

[B22] Friedman G. D. Udaltsova N. Habel L. A. (2011). Norepinephrine Antagonists and Cancer Risk. Int. J. Cancer 128 (3), 737–738. 10.1002/ijc.25351 20333678 PMC2993828

[B23] George A. J. Thomas W. G. Hannan R. D. (2010). The Renin-Angiotensin System and Cancer: Old Dog, New Tricks. Nat. Rev. Cancer 10 (11), 745–759. 10.1038/nrc2945 20966920

[B24] Giampieri R. Scartozzi M. Del Prete M. Faloppi L. Bianconi M. Ridolfi F. (2015). Prognostic Value for Incidental Antihypertensive Therapy with Beta-Blockers in Metastatic Colorectal Cancer. Medicine (Baltimore) 94 (24), e719. 10.1097/MD.0000000000000719 26091452 PMC4616528

[B25] Granados K. Hüser L. Federico A. Sachindra S. Wolff G. Hielscher T. (2020). T-type Calcium Channel Inhibition Restores Sensitivity to MAPK Inhibitors in De-differentiated and Adaptive Melanoma Cells. Br. J. Cancer 122 (7), 1023–1036. 10.1038/s41416-020-0751-8 32063604 PMC7109069

[B26] Greene A. S. Amaral S. L. (2002). Microvascular Angiogenesis and the Renin-Angiotensin System. Curr. Hypertens. Rep. 4 (1), 56–62. 10.1007/s11906-002-0054-x 11790293

[B27] Grimaldi-Bensouda L. Klungel O. Kurz X. de Groot M. C. Maciel Afonso A. S. de Bruin M. L. (2016). Calcium Channel Blockers and Cancer: a Risk Analysis Using the UK Clinical Practice Research Datalink (CPRD). BMJ Open 6 (1), e009147. 10.1136/bmjopen-2015-009147 PMC4716173 26747033

[B28] Grossman E. Messerli F. H. Goldbourt U. (2001). Antihypertensive Therapy and the Risk of Malignancies. Eur. Heart J. 22 (15), 1343–1352. 10.1053/euhj.2001.2729 11465967

[B29] Gupta A. K. Poulter N. R. Dobson J. Eldridge S. Cappuccio F. P. Caulfield M. (2010). Ethnic Differences in Blood Pressure Response to First and Second-Line Antihypertensive Therapies in Patients Randomized in the ASCOT Trial. Am. J. Hypertens. 23 (9), 1023–1030. 10.1038/ajh.2010.105 20725056

[B30] Hallas J. Christensen R. Andersen M. Friis S. Bjerrum L. (2012). Long Term Use of Drugs Affecting the Renin-Angiotensin System and the Risk of Cancer: a Population-Based Case-Control Study. Br. J. Clin. Pharmacol. 74 (1), 180–188. 10.1111/j.1365-2125.2012.04170.x 22243442 PMC3394143

[B31] Hicks B. M. Murray L. J. Powe D. G. Hughes C. M. Cardwell C. R. (2013). β-Blocker Usage and Colorectal Cancer Mortality: A Nested Case-Control Study in the UK Clinical Practice Research Datalink Cohort. Ann. Oncol. 24 (12), 3100–3106. 10.1093/annonc/mdt381 24050955

[B32] Holmes S. Griffith E. J. Musto G. Minuk G. Y. (2013). Antihypertensive Medications and Survival in Patients with Cancer: A Population-Based Retrospective Cohort Study. Cancer Epidemiol. 37 (6), 881–885. 10.1016/j.canep.2013.09.001 24075077

[B33] Ioannidis J. P. Zhou Y. Chang C. Q. Schully S. D. Khoury M. J. Freedman A. N. (2014). Potential Increased Risk of Cancer from Commonly Used Medications: an Umbrella Review of Meta-Analyses. Ann. Oncol. 25 (1), 16–23. 10.1093/annonc/mdt372 24310915 PMC3868319

[B34] Jansen L. Below J. Chang-Claude J. Brenner H. Hoffmeister M. (2012). Beta Blocker Use and Colorectal Cancer Risk: Population-Based Case-Control Study. Cancer 118 (16), 3911–3919. 10.1002/cncr.26727 22585669

[B35] Jansen L. Hoffmeister M. Arndt V. Chang-Claude J. Brenner H. (2014). Stage-specific Associations between Beta Blocker Use and Prognosis after Colorectal Cancer. Cancer 120 (8), 1178–1186. 10.1002/cncr.28546 24415516

[B36] Jansen L. Weberpals J. Kuiper J. G. Vissers P. A. J. Wolkewitz M. Hoffmeister M. (2017). Pre- and post-diagnostic Beta-Blocker Use and Prognosis after Colorectal Cancer: Results from a Population-Based Study. Int. J. Cancer 141 (1), 62–71. 10.1002/ijc.30717 28370155

[B37] Kanehira T. Tani T. Takagi T. Nakano Y. Howard E. F. Tamura M. (2005). Angiotensin II Type 2 Receptor Gene Deficiency Attenuates Susceptibility to Tobacco-specific Nitrosamine-Induced Lung Tumorigenesis: Involvement of Transforming Growth Factor-beta-dependent Cell Growth Attenuation. Cancer Res. 65 (17), 7660–7665. 10.1158/0008-5472.Can-05-0275 16140932

[B38] Lin C. S. Lin W. S. Lin C. L. Kao C. H. (2015). Carvedilol Use Is Associated with Reduced Cancer Risk: A Nationwide Population-Based Cohort Study. Int. J. Cardiol. 184, 9–13. 10.1016/j.ijcard.2015.02.015 25705003

[B39] Maeda H. Nakamura H. Fang J. (2013). The EPR Effect for Macromolecular Drug Delivery to Solid Tumors: Improvement of Tumor Uptake, Lowering of Systemic Toxicity, and Distinct Tumor Imaging *In Vivo* . Adv. Drug Deliv. Rev. 65 (1), 71–79. 10.1016/j.addr.2012.10.002 23088862

[B40] Mafiana R. N. Al-Kindi M. S. Mafiana N. Al Lawati A. S. Al Moundhri M. (2019). Impact of Metabolic Syndrome Diagnosis and its Treatment on Survival of Colorectal Cancer Patients. J. Cancer Epidemiol. 2019, 6527457. 10.1155/2019/6527457 31139216 PMC6500664

[B41] Makar G. A. Holmes J. H. Yang Y. X. (2014). Angiotensin-converting Enzyme Inhibitor Therapy and Colorectal Cancer Risk. J. Natl. Cancer Inst. 106 (2), djt374. 10.1093/jnci/djt374 24431411 PMC3952198

[B42] Mansouri D. McMillan D. C. Roxburgh C. S. Crighton E. M. Horgan P. G. (2013). The Impact of Aspirin, Statins and ACE-Inhibitors on the Presentation of Colorectal Neoplasia in a Colorectal Cancer Screening Programme. Br. J. Cancer 109 (1), 249–256. 10.1038/bjc.2013.292 23778525 PMC3708580

[B43] Mc Menamin Ú C. Murray L. J. Cantwell M. M. Hughes C. M. (2012). Angiotensin-converting Enzyme Inhibitors and Angiotensin Receptor Blockers in Cancer Progression and Survival: a Systematic Review. Cancer Causes Control 23 (2), 221–230. 10.1007/s10552-011-9881-x 22116540

[B44] Merai R. Siegel C. Rakotz M. Basch P. Wright J. Wong B. (2016). CDC Grand Rounds: A Public Health Approach to Detect and Control Hypertension. MMWR Morb Mortal Wkly Rep. 65 (45), 1261–1264. 10.15585/mmwr.mm6545a3 27855138

[B45] Michels K. B. Rosner B. A. Walker A. M. Stampfer M. J. Manson J. E. Colditz G. A. (1998). Calcium Channel Blockers, Cancer Incidence, and Cancer Mortality in a Cohort of U.S. Women: the Nurses' Health Study. Cancer 83 (9), 2003–2007. 10.1002/(sici)1097-0142(19981101)83:9<2003:aid-cncr17>3.0.co;2-3 9806660

[B46] Moher D. Liberati A. Tetzlaff J. Altman D. G. (2009). Preferred Reporting Items for Systematic Reviews and Meta-Analyses: the PRISMA Statement. Plos Med. 6 (7), e1000097. 10.1136/bmj.b2535 19621072 PMC2707599

[B47] Morris Z. S. Saha S. Magnuson W. J. Morris B. A. Borkenhagen J. F. Ching A. (2016). Increased Tumor Response to Neoadjuvant Therapy Among Rectal Cancer Patients Taking Angiotensin-Converting Enzyme Inhibitors or Angiotensin Receptor Blockers. Cancer 122 (16), 2487–2495. 10.1002/cncr.30079 27203227 PMC4998053

[B48] Numbere B. Fleming K. M. Walker A. Card T. R. (2015). Adrenergic Blockers and the Risk for Common Solid Cancers: a Case-Control Study. Eur. J. Cancer Prev. 26 (1), 86–93. 10.1097/cej.0000000000000218 26649549

[B49] Ogedegbe G. Shah N. R. Phillips C. Goldfeld K. Roy J. Guo Y. (2015). Comparative Effectiveness of Angiotensin-Converting Enzyme Inhibitor-Based Treatment on Cardiovascular Outcomes in Hypertensive Blacks versus Whites. J. Am. Coll. Cardiol. 66 (11), 1224–1233. 10.1016/j.jacc.2015.07.021 26361152 PMC4567693

[B50] Osumi H. Matsusaka S. Wakatsuki T. Suenaga M. Shinozaki E. Mizunuma N. (2015). Angiotensin II Type-1 Receptor Blockers Enhance the Effects of Bevacizumab-Based Chemotherapy in Metastatic Colorectal Cancer Patients. Mol. Clin. Oncol. 3 (6), 1295–1300. 10.3892/mco.2015.630 26807236 PMC4665652

[B51] Ozawa T. Hashiguchi Y. Yagi T. Fukushima Y. Shimada R. Hayama T. (2019). Angiotensin I-Converting Enzyme Inhibitors/angiotensin II Receptor Blockers May Reduce Tumor Recurrence in Left-Sided and Early Colorectal Cancers. Int. J. Colorectal Dis. 34 (10), 1731–1739. 10.1007/s00384-019-03379-y 31478086

[B52] Pahor M. Guralnik J. M. Ferrucci L. Corti M. C. Salive M. E. Cerhan J. R. (1996). Calcium-channel Blockade and Incidence of Cancer in Aged Populations. The Lancet 348 (9026), 493–497. 10.1016/s0140-6736(96)04277-8 8757150

[B53] Rosenberg L. Rao R. S. Palmer J. R. Strom B. L. Stolley P. D. Zauber A. G. (1998). Calcium Channel Blockers and the Risk of Cancer. J. Am. Med. Assoc. 279 (13), 1000–1004. 10.1001/jama.279.13.1000 9533498

[B54] Sipahi I. Debanne S. M. Rowland D. Y. Simon D. I. Fang J. C. (2010). Angiotensin-receptor Blockade and Risk of Cancer: Meta-Analysis of Randomised Controlled Trials. Lancet Oncol. 11 (7), 627–636. 10.1016/s1470-2045(10)70106-6 20542468 PMC4070221

[B55] Sorensen H. T. Olsen J. H. Mellemkjaer L. Thulstrup A. M. Steffensen F. H. McLaughlin J. K. (2000). Cancer Risk and Mortality in Users of Calcium Channel Blockers - A Cohort Study. Cancer 89 (1), 165–170. 10.1002/1097-0142(20000701)89:1<165:Aid-cncr21>3.0.Co;2-g 10897013

[B56] Stanford J. L. Martin E. J. Brinton L. A. Hoover R. N. (1986). Rauwolfia Use and Breast Cancer: a Case-Control Study. J. Natl. Cancer Inst. 76 (5), 817–822. 3457968

[B57] Sud S. O'Callaghan C. Jonker C. Karapetis C. Price T. Tebbutt N. (2018). Hypertension as a Predictor of Advanced Colorectal Cancer Outcome and Cetuximab Treatment Response. Curr. Oncol. 25 (6), e516–e526. 10.3747/co.25.4069 30607118 PMC6291274

[B58] Sung H. Ferlay J. Siegel R. L. Laversanne M. Soerjomataram I. Jemal A. (2021). Global Cancer Statistics 2020: GLOBOCAN Estimates of Incidence and Mortality Worldwide for 36 Cancers in 185 Countries. CA Cancer J. Clin. 71 (3), 209–249. 10.3322/caac.21660 33538338

[B59] Tang J. Li Z. Lu L. Cho C. H. (2013). β-Adrenergic System, a Backstage Manipulator Regulating Tumour Progression and Drug Target in Cancer Therapy. Semin. Cancer Biol. 23 (6 Pt B), 533–542. 10.1016/j.semcancer.2013.08.009 24012659

[B60] Tenenbaum A. Grossman E. Fisman E. Z. Adler Y. Boyko V. Jonas M. (2001). Long-term Diuretic Therapy in Patients with Coronary Disease: Increased colon Cancer-Related Mortality over a 5-year Follow-Up. J. Hum. Hypertens. 15 (6), 373–379. 10.1038/sj.jhh.1001192 11439311

[B61] Thomopoulos C. Parati G. Zanchetti A. (2015). Effects of Blood Pressure Lowering on Outcome Incidence in Hypertension: 4. Effects of Various Classes of Antihypertensive Drugs-Ooverview and Meta-Analyses. J. Hypertens. 33 (2), 195–211. 10.1097/hjh.0000000000000447 25485720

[B62] van der Knaap R. Siemes C. Coebergh J.-W. W. van Duijn C. M. Hofman A. Stricker B. H. C. (2008). Renin-anglotensin System Inhibitors, Angiotensin I-Converting Enzyme Gene Insertion/deletion Polymorphism, and Cancer. Cancer 112 (4), 748–757. 10.1002/cncr.23215 18181094

[B63] Wang K. L. Liu C. J. Chao T. F. Huang C. M. Wu C. H. Chen T. J. (2012). Long-term Use of Angiotensin II Receptor Blockers and Risk of Cancer: a Population-Based Cohort Analysis. Int. J. Cardiol. 167 (5), 2162–2166. 10.1016/j.ijcard.2012.05.096 22709730

[B64] Weberpals J. Jansen L. van Herk-Sukel M. P. P. Kuiper J. G. Aarts M. J. Vissers P. A. J. (2017). Immortal Time Bias in Pharmacoepidemiological Studies on Cancer Patient Survival: Empirical Illustration for Beta-Blocker Use in Four Cancers with Different Prognosis. Eur. J. Epidemiol. 32 (11), 1019–1031. 10.1007/s10654-017-0304-5 28864947

[B65] Zhou Q. Chen D. S. Xin L. Zhou L. Q. Zhang H. T. Liu L. (2020). The Renin-Angiotensin System Blockers and Survival in Digestive System Malignancies: A Systematic Review and Meta-Analysis. Medicine (Baltimore) 99 (7), e19075. 10.1097/MD.0000000000019075 32049809 PMC7035076

