# Supplementary material for: Effects of Antihypertensive Drugs Use on Risk and Prognosis of Colorectal Cancer: A Meta-Analysis of 37 Observational Studies
Source: Front Pharmacol. 2022 Jan 11;12:670657. doi: 10.3389/fphar.2021.670657 (PMC8789244; doi:10.3389/fphar.2021.670657)
Supplement: Supplementary file 1 [file Table4.doc]

| Study | Selection | Comparability | Outcome | Total |
| --- | --- | --- | --- | --- |
| Incidence--cohort study | | | | |
| B.N AB, et al 2003 | 4 | 1 | 3 | 8 |
| Brasky, T. M., et al 2021 | 4 | 1 | 3 | 8 |
| Chang, et al 2015 | 3 | 1 | 3 | 7 |
| Cheung, KS, et al 2020 | 3 | 2 | 3 | 8 |
| G.B, et al 2016 | 4 | 1 | 3 | 8 |
| Lin, et al, et al 2015 | 4 | 1 | 3 | 8 |
| Makar GA, et al 2014 | 4 | 1 | 2 | 7 |
| Mansouri, et al 2013 | 4 | 1 | 2 | 7 |
| Michels K B, et al 1998 | 4 | 1 | 3 | 8 |
| Pahor M, et al 1996 | 3 | 1 | 3 | 7 |
| Tenenbaum, et al 2001 | 4 | 1 | 3 | 8 |
| van der Knaap R, et al 2008 | 3 | 1 | 3 | 7 |
| Wang, et al 2012 | 4 | 1 | 3 | 8 |
| Prognosis--cohort study | | | | |
| Ahl R, et al 2020 | 3 | 1 | 2 | 6 |
| Cardwell CR, et al 2014 | 4 | 1 | 2 | 7 |
| Cui Y, er al 2019 | 4 | 1 | 3 | 8 |
| Fiala O, et al 2019 | 3 | 1 | 3 | 7 |
| Giampieri, et al 2015 | 3 | 1 | 2 | 6 |
| Hicks B M, et al 2013 | 4 | 1 | 3 | 8 |
| Holmes S, et al 2013 | 4 | 1 | 2 | 7 |
| Jansen, et al 2014 | 3 | 1 | 3 | 7 |
| Jansen, et al 2017 | 4 | 1 | 3 | 8 |
| Mafiana RN, et al 2019 | 2 | 1 | 3 | 6 |
| Morris, et al 2016 | 3 | 1 | 3 | 7 |
| Osumi H, et al 2015 | 3 | 1 | 3 | 7 |
| Sorensen, et al 2000 | 4 | 1 | 3 | 8 |
| Weberpals J, et al 2017 | 4 | 1 | 2 | 7 |
| Bowles EJA, et al 2019 | 4 | 1 | 3 | 8 |
| Ozawa T, et al 2019 | 3 | 1 | 3 | 7 |
| Sud S, et al 2018 | 3 | 1 | 2 | 6 |

**Supplementary table 4.** Quality assessment of eligible studies by Newcastle-Ottawa Scale.

**Supplementary table 4.** Quality assessment of eligible studies by Newcastle-Ottawa Scale.

| Study | Selection | Comparability | Exposure | Total |
| --- | --- | --- | --- | --- |
| Incidence--case control study | | | | |
| Boudreau DM, et al 2008 | 3 | 1 | 2 | 6 |
| Dierssen Sotos T, et al 2017 | 4 | 1 | 2 | 7 |
| Friedman GD, et al 2011 | 3 | 1 | 2 | 6 |
| Hallas J, et al 2012 | 4 | 1 | 2 | 7 |
| Jansen, et al 2012 | 4 | 1 | 3 | 8 |
| Numbere B, et al 2015 | 4 | 1 | 3 | 8 |
| Rosenberg L, et al 1998 | 3 | 1 | 3 | 7 |
