# Supplementary figures and images for: Effects of Antihypertensive Drugs Use on Risk and Prognosis of Colorectal Cancer: A Meta-Analysis of 37 Observational Studies
Source: Front Pharmacol. 2022 Jan 11;12:670657. doi: 10.3389/fphar.2021.670657 (PMC8789244; doi:10.3389/fphar.2021.670657)

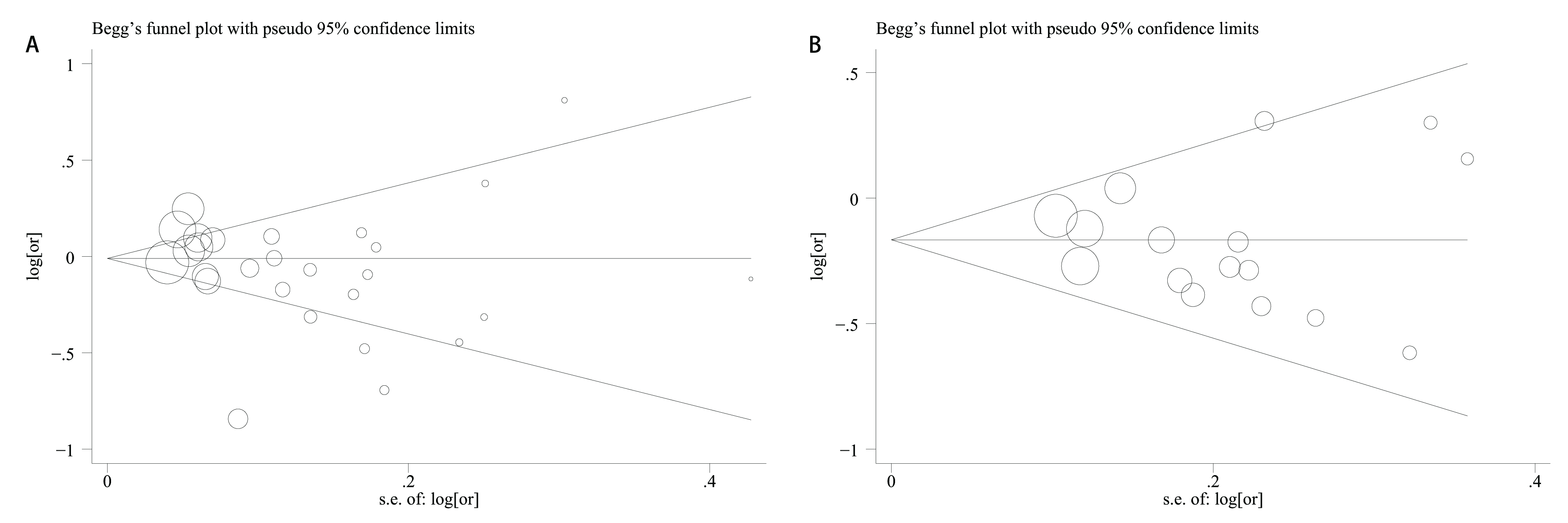

Supplement: Supplementary file 2 [file Image6.TIF]

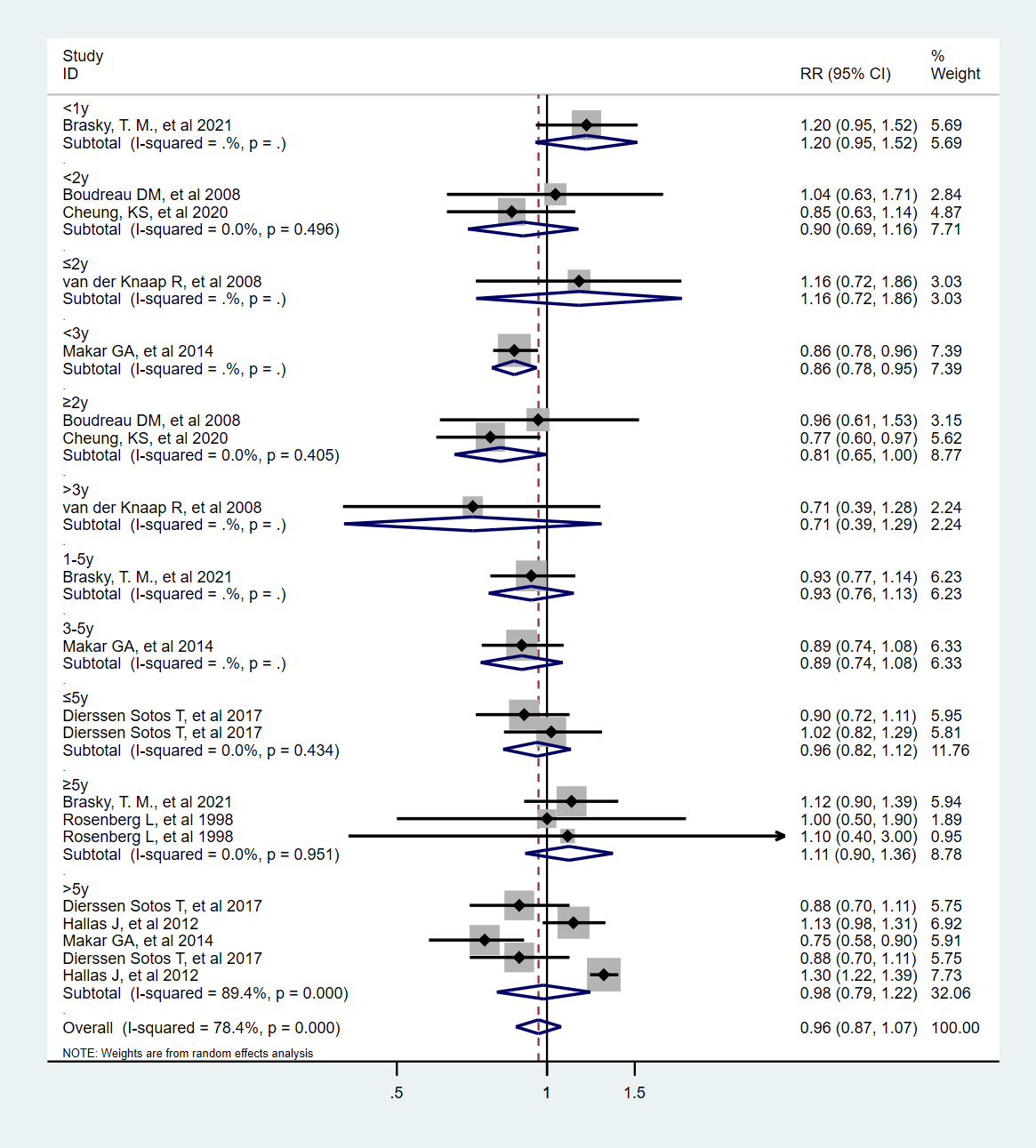

Supplement: Supplementary file 3 [file Image3.TIF]

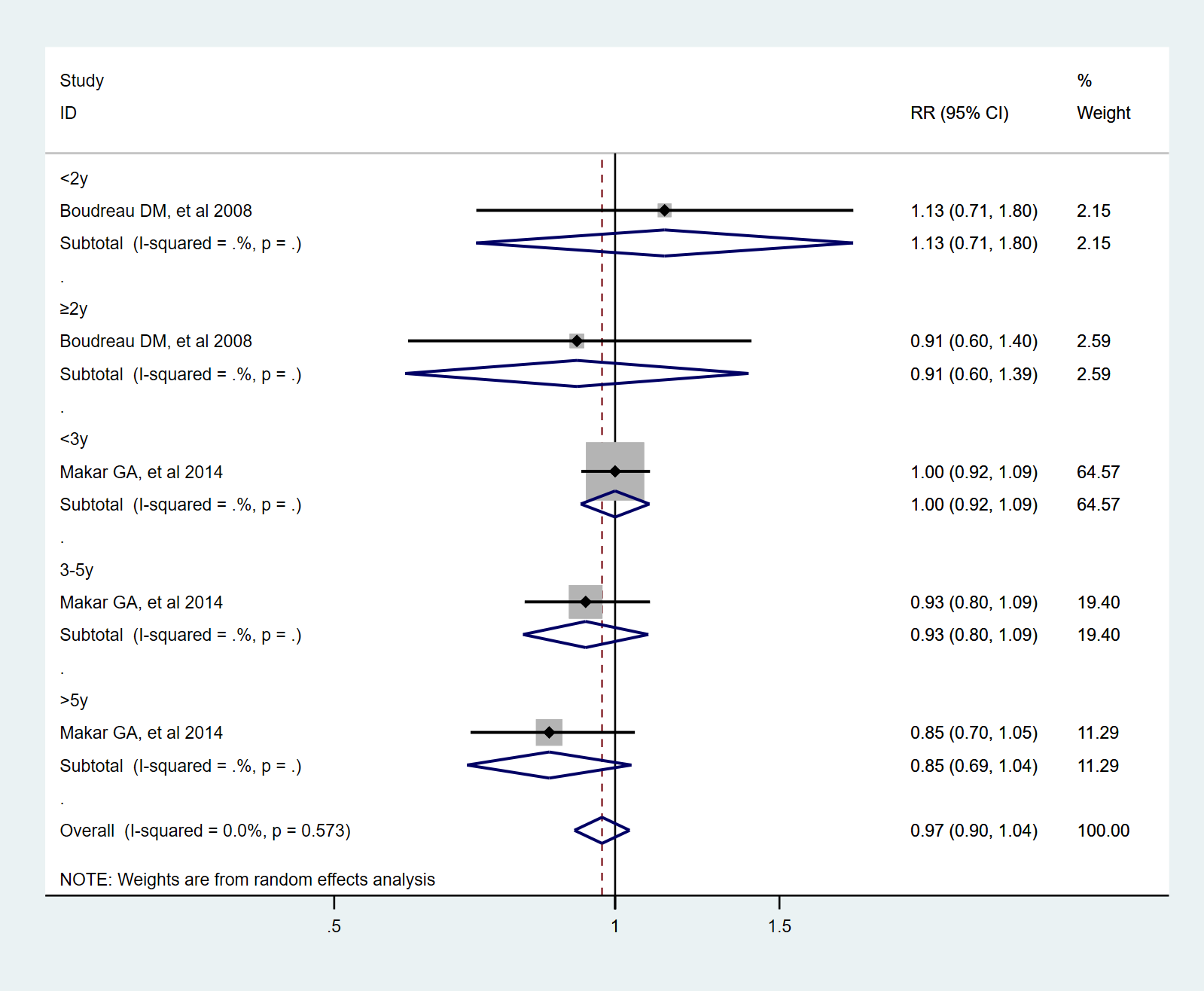

Supplement: Supplementary file 4 [file Image4.TIF]

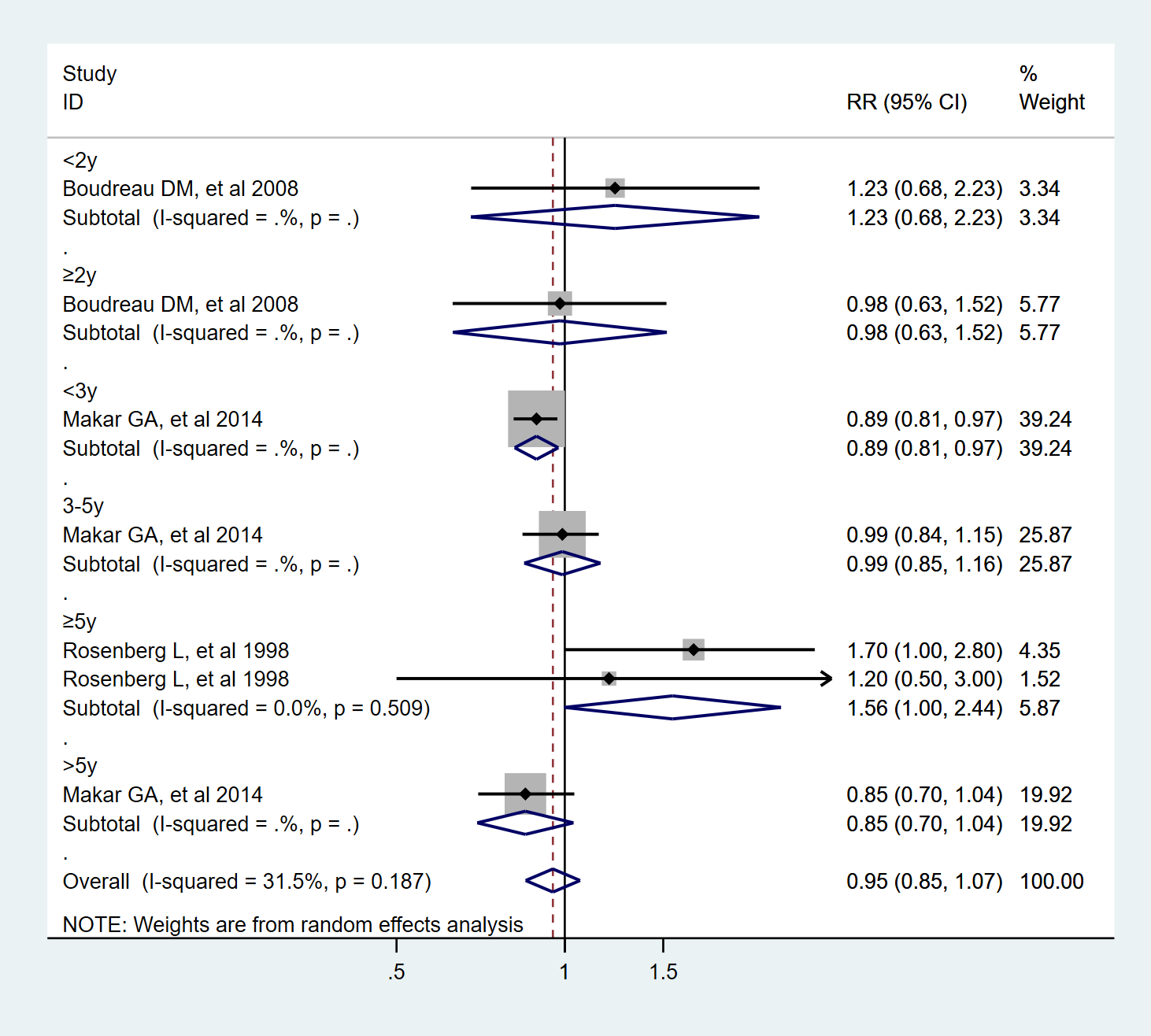

Supplement: Supplementary file 5 [file Image2.TIF]

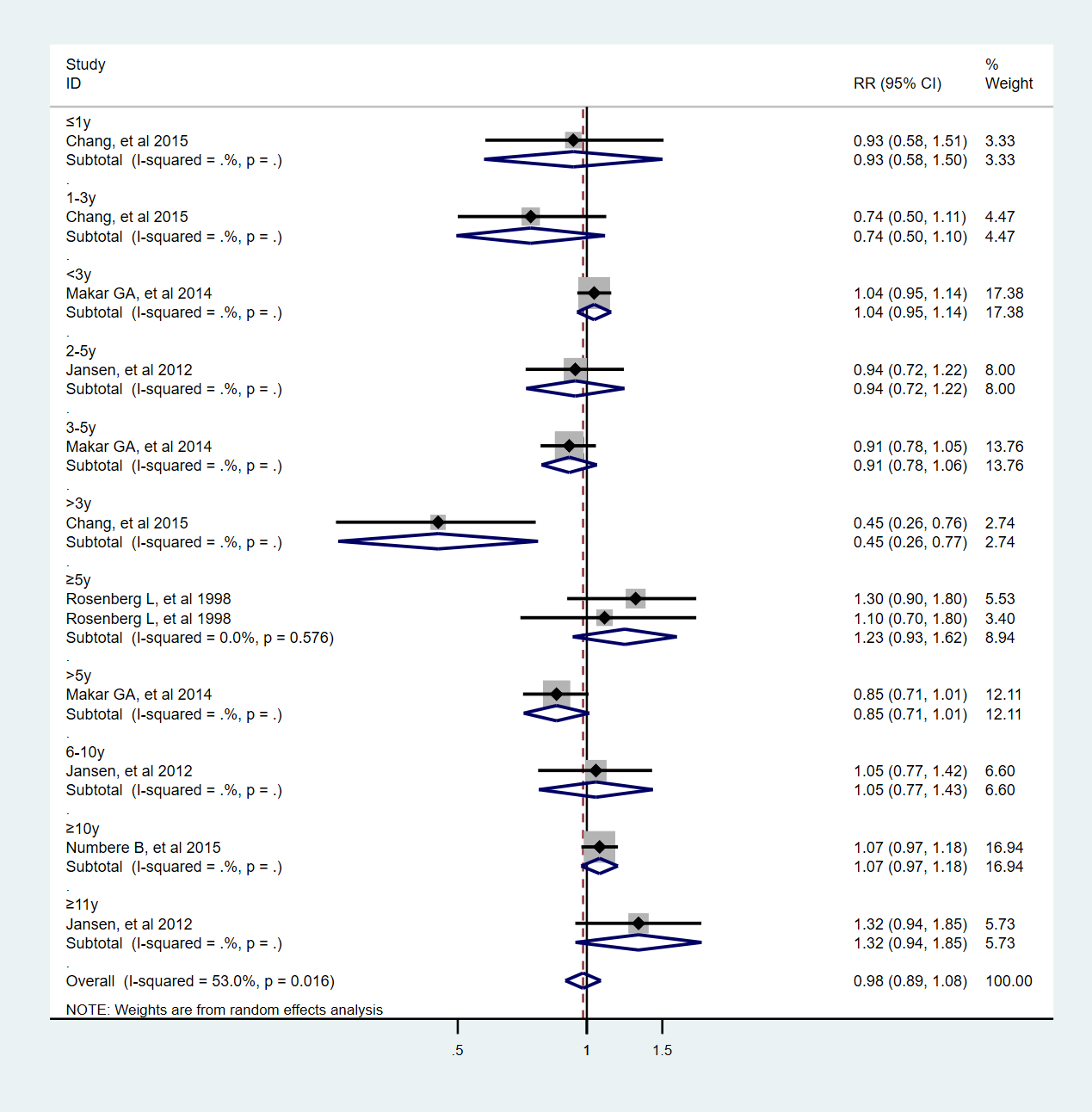

Supplement: Supplementary file 6 [file Image1.TIF]

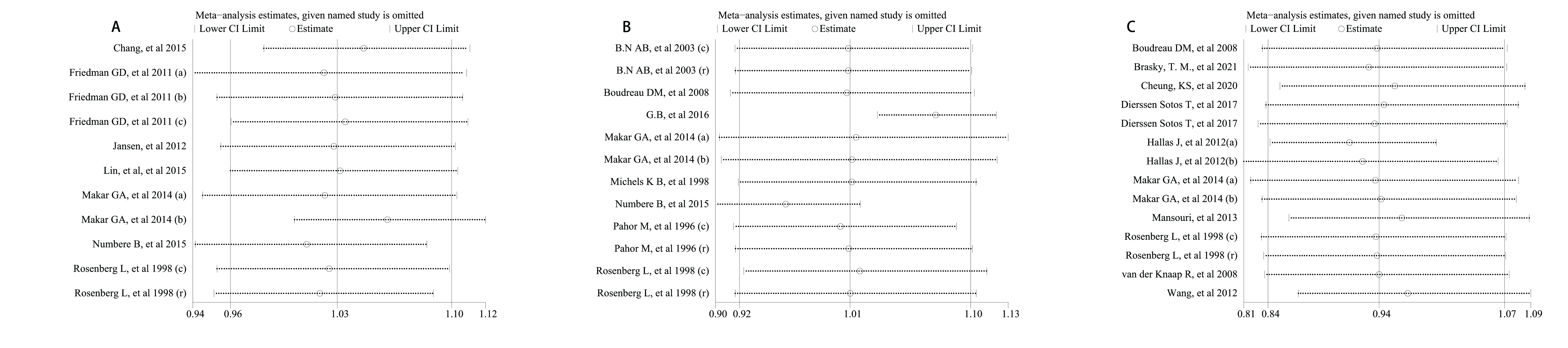

Supplement: Supplementary file 7 [file Image7.TIF]

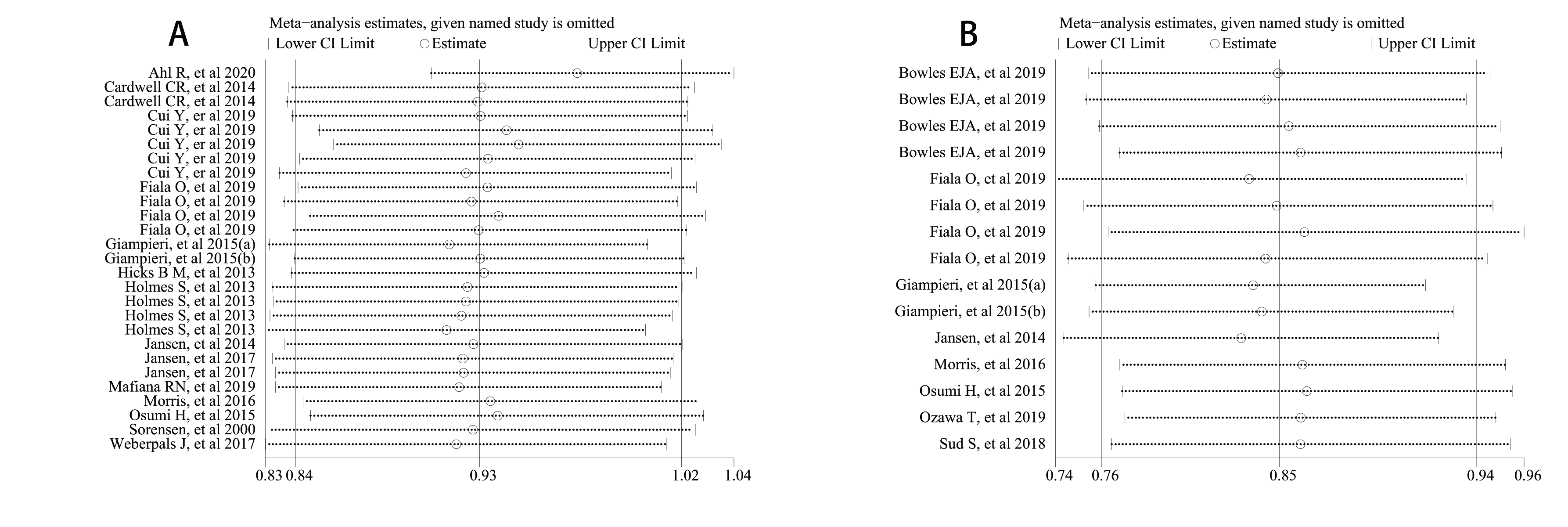

Supplement: Supplementary file 11 [file Image8.TIF]

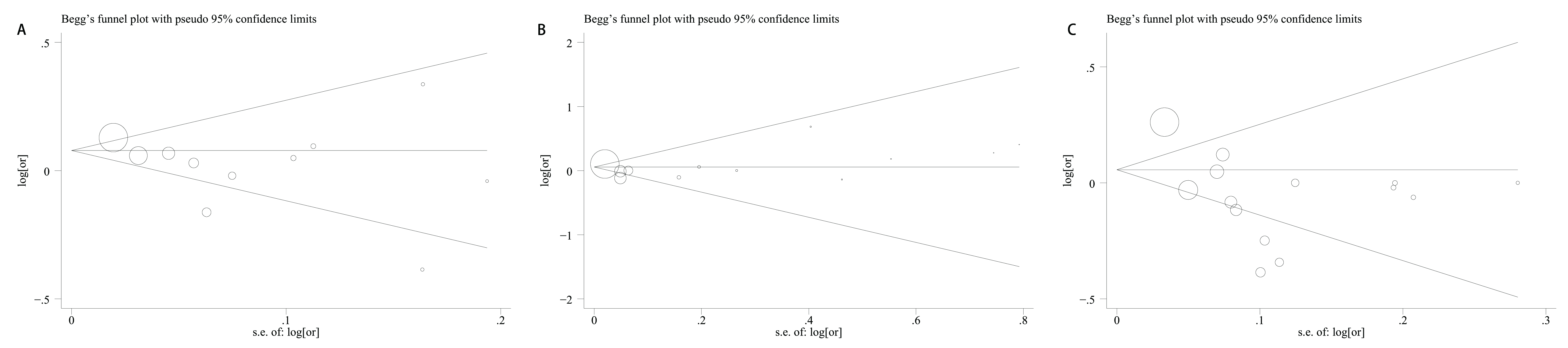

Supplement: Supplementary file 12 [file Image5.TIF]
