# Supplementary material for: Effects of Antihypertensive Drugs Use on Risk and Prognosis of Colorectal Cancer: A Meta-Analysis of 37 Observational Studies
Source: Front Pharmacol. 2022 Jan 11;12:670657. doi: 10.3389/fphar.2021.670657 (PMC8789244; doi:10.3389/fphar.2021.670657)
Supplement: Supplementary file 9 [file Table2.DOCX]

**Table S2. Search strategy**

| **Databases searched**  PubMed, Web of Science, Embase, Cochrane Library |
| --- |
| All searches limited to human studies. No date or language restrictions. |
| **Search terms**  #1 “Cancer” or “Carcinoma” or “Malignancy” or “Neoplasm” or “Tumor”  #2 “rectum” or “colon” or “colorectal”  #3 “Antihypertensive Drug” or “Antihypertensive Agent”  #4 “ARBs” or “ARB” or “Angiotensin Receptor Antagonists” or “Angiotensin Receptor Blocker” or “Angiotensin-Receptor Blocker” or “Angiotensin Receptor Blockers” or “Angiotensin Receptor Antagonist” or “Angiotensin II Receptor Blocker” or “Angiotensin II Receptor Blockers” or “Angiotensin Receptor Blockade” or “Renin Angiotensin System Inhibitor” or “Renin-Angiotensin System Inhibitor” or “RAS Inhibitor” or “Candesartan” or “Eprosartan” or “Irbesartan” or “Losartan” or “Olmesartan” or “Tasosartan” or “Telmisartan” or “Valsartan”  #5 “ACEI” or “ACEIs” or “Angiotensin-Converting Enzyme Inhibitors” or “Angiotensin Converting Enzyme Inhibitor” or “ACE Inhibitor” or “Benazepril” or “Captopril” or “Delapril” or “Ramipril” or “Cilazapril” or “Enalapril” or “Fosinopril” or “Perindopril” or “Imidapril” or “Lisinopril” or “Moexipril” or “Quinapril” or “Trandolapril” or “Spirapril” or “Temocapril” or “Zofenopril”  #6 “Calcium Channel Blockers” or “Calcium Channel Blocker” or “Calcium Channel Antagonists” or “Calcium Channel Antagonist” or “Amlodipine” or “Benidipine” or “Diltiazem” or “Felodipine” or “Isradipine” or “Manidipine” or “Nicardipine” or “Nifedipine” or “Nisoldipine” or “Nitrendipine” or “Verapamil”  #7 “Beta Blockers” or “Beta-Blocker” or “Beta-Receptor Antagonist” or “Beta Adrenergic Antagonists” or “Acebutolol” or “Atenolol” or “Bisoprolol” or “Carvedilol” or “Celiprolol” or “Esmolol” or “Labetalol” or “Metoprolol” or “Nadolol” or “Nebivalol” or “Propanolol” or “Sotalol” or “Timolol”  #8 “Diuretics” or “Chlorothiazide” or “Hydrochlorothiazide” or “Bendroflumethiazide” or “Hydroflumethiazide” or “Methylchlothiazide” or “Polythiazide” or “Trichlormethiazide” or “Chlorthalidone” or “Metolazone” or “Indapamide” #9 “incidence” or “prognoses” or “prognosis” or “prognostic” or “survival” or “mortality”#10 #1 AND #2#11 #3 OR #4 OR #5 OR #6 OR #7 OR #8#12 #9 AND #10 AND #11 |
