# Supplementary material for: Effects of Antihypertensive Drugs Use on Risk and Prognosis of Colorectal Cancer: A Meta-Analysis of 37 Observational Studies
Source: Front Pharmacol. 2022 Jan 11;12:670657. doi: 10.3389/fphar.2021.670657 (PMC8789244; doi:10.3389/fphar.2021.670657)
Supplement: Supplementary file 10 [file Table3.docx]

**Supplementary table 3.** Reasons of excluded studies.

| **Study** | **No** | **Title** | **Reason for Exclusion** |
| --- | --- | --- | --- |
| Zhou, Q.,et al. (2020). | 1 | The renin-angiotensin system blockers and survival in digestive system malignancies: A systematic review and meta-analysis. | Meta-analysis |
| Asgharzadeh, F.,et al. (2018). | 2 | The Therapeutic Potential of Angiotensin-converting Enzyme and Angiotensin Receptor Inhibitors in the Treatment of Colorectal Cancer: Rational Strategies and Recent Progress. | Review |
| Ishikane, S., et al. (2018). | 3 | The role of angiotensin II in cancer metastasis: Potential of renin-angiotensin system blockade as a treatment for cancer metastasis. | Review |
| Song, T.,et al. (2017). | 4 | The effect of angiotensin system inhibitors (angiotensin-converting enzyme inhibitors or angiotensin receptor blockers) on cancer recurrence and survival: a meta-analysis | Meta-analysis |
| Long, L.,et al. (2017). | 5 | Association of ACEIs/ARBs therapy with digestive system neoplasms: A meta-analysis. | Meta-analysis |
| Dai, Y. N.,et al. (2015). | 6 | Angiotensin-converting enzyme inhibitors/angiotensin receptor blockers therapy and colorectal cancer: a systematic review and meta-analysis. | Meta-analysis |
| Dézsi, C. A.,et al. (2014). | 7 | A review of clinical studies on angiotensin II receptor blockers and risk of cancer. | Review |
| Mc Menamin Ú, C.,et al. (2012). | 8 | Angiotensin-converting enzyme inhibitors and angiotensin receptor blockers in cancer progression and survival: A systematic review. | Meta-analysis |
| Grossman, E., et al. (2002). | 9 | Carcinogenicity of antihypertensive therapy. | Review |
| Pahor, M., et al. (1998). | 10 | Is the use of some calcium antagonists linked to cancer? Evidence from recent observational studies. | Review |
| Sanyal, S., et al. (2019). | 11 | ACE Inhibitor Therapy Does Not Influence the Survival Outcomes of Patients with Colorectal Liver Metastases Following Liver Resection. | No available data |
| Htoo, P. T., et al. (2019). | 12 | ACEI or angiotensin receptor blockers (ARB) versus guideline-recommended clinical alternatives (beta blockers, calcium channel blockers [CCB], and thiazides). | Compared with antihypertensive drugs |
| Ahl, R., et al. (2019). | 13 | The Relationship Between Severe Complications, Beta-Blocker Therapy and Long-Term Survival Following Emergency Surgery for Colon Cancer. | No available data |
| González Ruiz, M. A., et al. (2018). | 14 | Effect of anti-hypertension therapy in colorectal carcinoma patients. | Conference abstract |
| Boas, F. E., et al. (2017). | 15 | Angiotensin receptor blockers and the risk of cancer: data mining of a spontaneous reporting database and a claims database. | Not about colorectal cancer |
| Sud, S., et al. (2016). | 16 | Adjuvant Medications That Improve Survival after Locoregional Therapy. | Not about colorectal cancer |
| Htoo, P. T., et al. (2015). | 17 | Hypertension and beta-blocker use as prognostic and predictive factors in metastatic colorectal cancer: A retrospective analysis of NCIC CTG CO.17. | Conference abstract |
| Tuazon, S. A., et al. (2014). | 18 | Angiotensin converting enzyme inhibitor and angiotensin receptor blocker use and outcomes in patients with colorectal cancer. | Conference abstract |
| Musselman, R. P., et al. (2014). | 19 | Association between beta blocker usage and cancer survival in a large, matched population study among hypertensive patients. | Conference abstract |
| Grimaldi-Bensouda, L., et al. (2014). | 20 | Calcium channel blockers and cancer risk using the UK CPRD. | Repeat study |
| Deshpande, G., et al. (2014). | 21 | Association between cardiovascular drugs and colon cancer. | Conference abstract |
| Engineer, D. R., et al. (2013). | 22 | Exposure to ACEI/ARB and beta-Blockers Is Associated with Improved Survival and Decreased Tumor Progression and Hospitalizations in Patients with Advanced Colon Cancer. | No available data |
| Panigadi, G. N., et al. (2012). | 23 | Relationship between colorectal neoplasia and angiotensin receptor blockers and angiotensin-converting enzime inhibitor. | Conference abstract |
| Magliano, D. J., et al. (2012). | 24 | Incidence and predictors of all-cause and site-specific cancer in type 2 diabetes: the Fremantle Diabetes Study. | Diabetic, not hypertension |
| Hesse, U., et al. (2012). | 25 | Calcium channel blocker (CCB) treatments and cancer risk: Linking danish national health care databases. | Conference abstract |
| Engineer, D. R., et al. (2012). | 26 | Exposure to beta-blockers and ACEI/ARB is associated with improved survival in patients with advanced colon cancer. | No available data |
| Bhaskaran, K., et al. (2012). | 27 | Angiotensin receptor blockers and risk of cancer: cohort study among people receiving antihypertensive drugs in UK General Practice Research Database. | Compared with antihypertensive drugs |
| Azoulay, L., et al. (2012). | 28 | Long-term use of angiotensin receptor blockers and the risk of cancer. | Compared with antihypertensive drugs |
| Shah, S. M., et al. (2011). | 29 | Does β-adrenoceptor blocker therapy improve cancer survival? Findings from a population-based retrospective cohort study. | Compared with antihypertensive drugs |
| Chang, C. H., et al. (2011). | 30 | Angiotensin receptor blockade and risk of cancer in type 2 diabetes mellitus: a nationwide case-control study. | Diabetic, not hypertension |
| Bhaskaran, K., et al. (2011). | 31 | The risk of cancer associated with angiotensin-II receptor blockers. | Compared with antihypertensive drugs |
| Friedman, G. D., et al. (2009). | 32 | Screening pharmaceuticals for possible carcinogenic effects: initial positive results for drugs not previously screened. | No available data |
| Khurana, V., et al. (2005). | 33 | Angiotensin Converting Enzyme (ACE) inhibitors reduce the incidence of colon cancer: A study of half a million US veterans. | Conference abstract |
| Robertson, D. J., et al. (2004). | 34 | The effect of beta-blockers on incident colorectal adenomas. | Conference abstract |
| Tenenbaum, A., et al. (2001). | 35 | Aspirin can reduce risk for colon but not renal cancer in coronary patients on diuretics | No available data |
| Tenenbaum, A., et al. (2001). | 36 | Is diuretic therapy associated with an increased risk of colon cancer? | No available data |
| Lindholm, Lars H.,et al. (2001). | 37 | Relation between drug treatment and cancer In hypertenslves in the Swedish Trial in Old Patients with Hypertension 2: a 5-year, prospective, randomised, controlled trial. | SIR as effect value |
| Friis, S.,et al. (2001). | 38 | Angiotensin-converting enzyme inhibitors and the risk of cancer: a population-based cohort study in Denmark. | SIR as effect value |
| Sorensen, H. T.,et al. (2000). | 39 | Cancer risk and mortality in users of calcium channel blockers - A cohort study. | SIR as effect value |
